# Reciprocal regulation of Solo and Src orchestrates Src trafficking to promote mesenchymal cell migration

**DOI:** 10.1016/j.isci.2025.112618

**Published:** 2025-05-09

**Authors:** Florian Meyer, Cristiana Lungu, Bettina Noll, David Benz, Felix Fränkle, Miguel Â. Ferreira, Raluca Tamas, Monilola A. Olayioye

**Affiliations:** 1 University of Stuttgart, Institute of Cell Biology and Immunology, 70569 Stuttgart, Germany; 2 University of Stuttgart, Stuttgart Research Center Systems Biology, 70569 Stuttgart, Germany

**Keywords:** Biochemistry, Cell biology

## Abstract

Rho GTPases are key regulators of cell motility and membrane trafficking, influencing critical processes such as epithelial-mesenchymal transition (EMT). Among them, the small GTPase RhoB plays a pivotal role, but the mechanisms underlying its regulation remain largely unclear. We have previously identified the Rho guanine nucleotide exchange factor (RhoGEF) Solo (ARHGEF40) as a regulator of endosomal RhoB in epithelial cells. Here, we find that Solo is upregulated in breast cancer cells with high EMT scores and promotes cell motility through its RhoGEF activity. Solo’s ability to enhance migration is further regulated by phosphorylation at tyrosine 242, mediated by the proto-oncogene Src. By combining high-resolution imaging with photoconversion assays, we further demonstrate that Solo regulates Src trafficking dynamics, localization, and consequently signaling at focal adhesions. Together, our data identify Solo as a novel feedback regulator of Src and a key driver of the motility of breast cancer cells with mesenchymal characteristics.

## Highlights

• Solo expression is increased in breast cancer cells with high EMT signatures
• Solo promotes breast cancer cell migration in a RhoGEF-dependent manner
• Src phosphorylates Solo at Y242 and cooperates with Solo to promote cell migration
• Loss of Solo disrupts Src trafficking and focal adhesion signaling dynamics

## Graphical abstract

**Figure:**
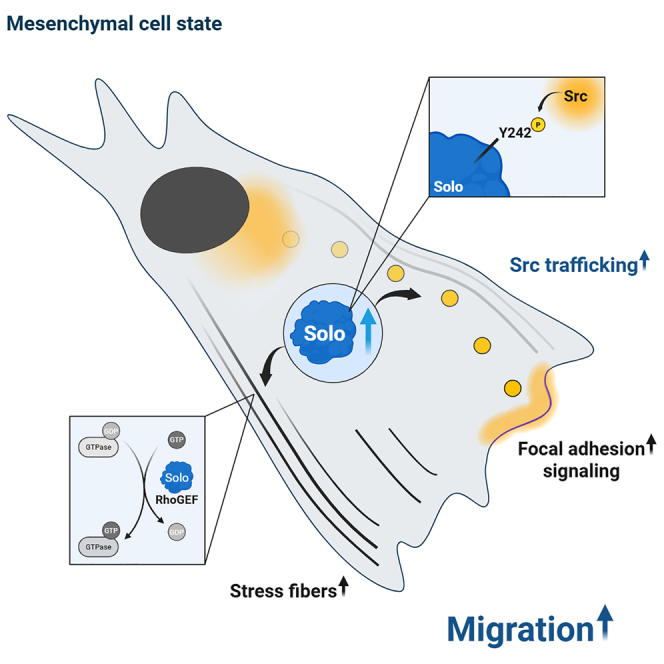

## Introduction

Epithelial-mesenchymal transition (EMT) is a central process in both development and disease, particularly in cancer metastasis where it endows epithelial cells with enhanced migratory and invasive properties.^1^ Central to this transition is the extensive remodeling of the actin cytoskeleton, along with the coordinated redistribution of signaling proteins through membrane trafficking. Rho GTPases are key molecular players that orchestrate these cellular processes.^2^ Acting as molecular switches, Rho GTPases cycle between an active GTP-bound state and an inactive GDP-bound state. This cycling is controlled by guanine nucleotide exchange factors (GEFs), which promote the exchange of GDP for GTP to activate the GTPases, and GTPase-activating proteins (GAPs), which stimulate the intrinsic GTPase activity to inactivate them. In addition to mutational activation of the GTPases, the activity or expression of their regulatory GEFs and GAPs is often altered in cancer. This emphasizes the need to better understand these regulators.^3^

Among Rho GTPases, RhoB plays a critical role in modulating intracellular trafficking and cytoskeletal dynamics, impacting cell shape, adhesion, and motility,^4^ also in the context of EMT.^5^ Unlike RhoA and RhoC, RhoB is unique in its ability to localize to endocytic compartments, where it regulates the trafficking of key signaling molecules such as the EGF receptor (EGFR).^6^ Additionally, RhoB controls the stimulus-induced peripheral delivery and activation of the proto-oncogene Src.^7^ Despite these critical functions, the regulation of RhoB remains poorly understood, with only a few GEFs identified as established activators such as GEF-H1 or ARHGEF10.^8^^,^^9^ Recently, our work has identified the GEF protein Solo, also known as ARHGEF40, to regulate endosomal RhoB and the EGFR signaling pathway in epithelial cells,^10^ in addition to the role of Solo in RhoA activation.^11^^,^^12^ However, the mechanisms governing the regulation of Solo have not been explored to date.

In this study, analysis of TCGA breast cancer data and a panel of breast cancer cell lines revealed that Solo is upregulated in cells with a high EMT score, acting as a positive regulator of cell motility. Solo upregulation was sufficient to promote the migration of epithelial cells, an effect dependent on its RhoGEF activity. This process was furthermore modulated by Src-induced tyrosine phosphorylation of Solo at Y242. Interestingly, Solo in turn regulated Src trafficking to the cell periphery, ensuring proper downstream signaling at focal adhesions. These insights suggest that Solo, a novel feedback regulator of Src, is a key mediator of the cellular changes associated with the mesenchymal cell state.

## Results

### Solo is upregulated in cells with mesenchymal traits

**Figure 1:**
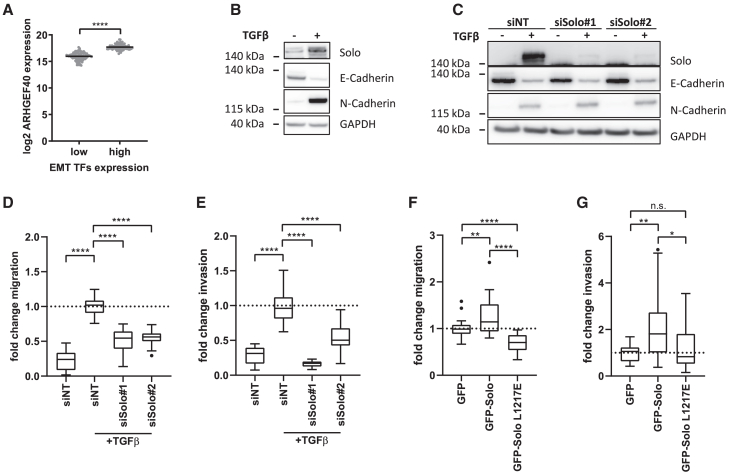
Solo is upregulated in EMT and regulates TGFβ induced cell motility in MCF10A cells (A) *ARHGEF40/Solo* gene expression in a cohort of TCGA BRCA patients with high versus low epithelial–mesenchymal transition transcription factors (EMT TFs; *SLUG, ZEB1, ZEB2* and *TWIST2*) quartile expression. Dots represent samples and the line indicates the mean. Statistical significance was determined by a two-tailed unpaired t-test with Welch’s correction. (B) Solo, E-Cadherin and N-Cadherin protein levels in MCF10A cells treated for 7 days with 10 ng/mL TGFβ (+) or left untreated (−). Lysates were prepared and analyzed by immunoblotting with the indicated antibodies (see also Figure S1), *n* = 3. GAPDH served as loading control. (C) Following 7 days of TGFβ treatment, MCF10A cells were transfected with either control (siNT) or two independent Solo specific siRNAs. Cell lysates were isolated 72 h later. Cells without TGFβ treatment were used as a reference. The lysates were analyzed by immunoblotting with the indicated antibodies. GAPDH served as loading control, *n* = 1. Transwell migration (D, 6 h) and invasion assays (E, 24 h) toward a serum gradient were performed on TGFβ-treated MCF10A cells 72 h after Solo depletion by two independent siRNAs. Cells without TGFβ treatment were used as a reference. siNT transfected cells served as control and were used for data normalization. The latter was set to 1 and indicated with the dotted line. Data shown as Tukey boxplots with each dot representing one field of view. Statistical significance was determined by Brown-Forsythe and Welch ANOVA with Games-Howell’s multiple comparisons (E, *N* = 27–29), *n* = 3. Transwell migration (F, 6 h) and invasion assays (G, 24 h) toward a serum gradient were performed in the GFP-Solo MCF10A model system expressing wild-type or catalytically inactive GFP-Solo L1217E (see also Figure S3). Cells expressing free GFP served as control and were used for data normalization. The latter was set to 1 and indicated with the dotted line. Data shown as Tukey boxplots with each dot representing one field of view. Statistical significance was determined by Brown-Forsythe and Welch ANOVA with Games-Howell’s multiple comparisons, *n* = 3, *N* = 27–31 for (F), *N* = 21–25 for (G). (D–G) Tukey boxplots range from the 25th to 75th percentile with a center line indicating the median. Whiskers extend 25th and 75th percentile by 1.5x inter-quartile distance, outliers are represented by dots. ∗∗∗∗*p* < 0.0001, ∗∗∗*p* < 0.001, ∗∗*p* < 0.01, ∗*p* < 0.05, n.s. not significant.

To explore the potential association between the RhoGEF Solo and EMT, we analyzed *ARHGEF40/Solo* gene expression in a TCGA breast cancer dataset. To this end, the expression data were categorized into high and low EMT transcription factor expression groups based on the levels of *SLUG*, *ZEB1*, *ZEB2*, and *TWIST*. Notably, *Solo* expression was significantly elevated in patients with high EMT transcription factor expression (Figure 1A). This observation was further supported by *in vitro* studies using the non-transformed breast epithelial cell line MCF10A. When treated with TGFβ, these cells undergo an EMT-like process characterized by upregulation of mesenchymal markers such as N-Cadherin and downregulation of epithelial markers like E-Cadherin^13^^,^^14^ (Figure 1B). Consistent with the findings from the TCGA analysis, Solo transcript and protein levels were strongly upregulated following TGFβ treatment (Figures S1 and 1B). Together, these data hint at a role of Solo in the mesenchymal cell state.

### Solo is a positive regulator of cell motility in MCF10A cells

To explore the role of Solo in the mesenchymal cell state, we knocked down Solo using two independent, previously validated siRNAs,^10^ in MCF10A cells pre-treated with TGFβ for 7 days. E− and N-Cadherin levels, assessed via western blotting, served as EMT markers. The cadherin switching behavior was comparable between control and Solo-depleted cells (Figure 1C), indicating that Solo is not required for the maintenance of the mesenchymal state.

EMT is associated with increased cell motility, a process regulated by Rho GTPase activity.^4^ Since Solo acts as a RhoGEF for RhoA^11^^,^^12^ and RhoB,^10^ we hypothesized that the elevated level of Solo could promote motility in the mesenchymal cell state. To test this, we first validated that Solo depletion reduces RhoA activity under our experimental conditions (Figure S2A). Next, we performed transwell migration and invasion assays on TGFβ-treated MCF10A cells with and without Solo depletion. Notably, Solo depletion reduced the migration of TGFβ-treated MCF10A cells by approximately 40% and invasion by 70% (Figures 1D and 1E), suggesting that Solo facilitates cell motility under these conditions.

We next examined whether Solo expression alone is sufficient to enhance the motility of MCF10A cells. For this, we generated MCF10A cells stably expressing GFP-Solo in a doxycycline-controllable manner. GFP-Solo-expressing cells exhibited significantly increased migration and invasion compared to control cells expressing GFP alone (Figures S3, 1F, and 1G). This was strictly dependent on the RhoGEF activity of Solo, as cells expressing the catalytically inactive variant L1217E^12^ showed significantly reduced motility compared to those expressing the wild-type protein (Figures 1F and 1G). Indeed, expression of wild-type Solo led to a 2-fold increase of active RhoA levels (Figure S2B). These results demonstrate that Solo is sufficient to enhance MCF10A cell motility in a RhoGEF-dependent manner. These results were validated in HeLa cells, in which we previously demonstrated that Solo functions as a GEF for RhoA/B.^10^ Specifically, depletion of Solo reduced cell motility (Figure S4A), whereas its overexpression enhanced motility (Figure S4B).

### Solo is phosphorylated on Y242 by the proto-oncogene Src

**Figure 2:**
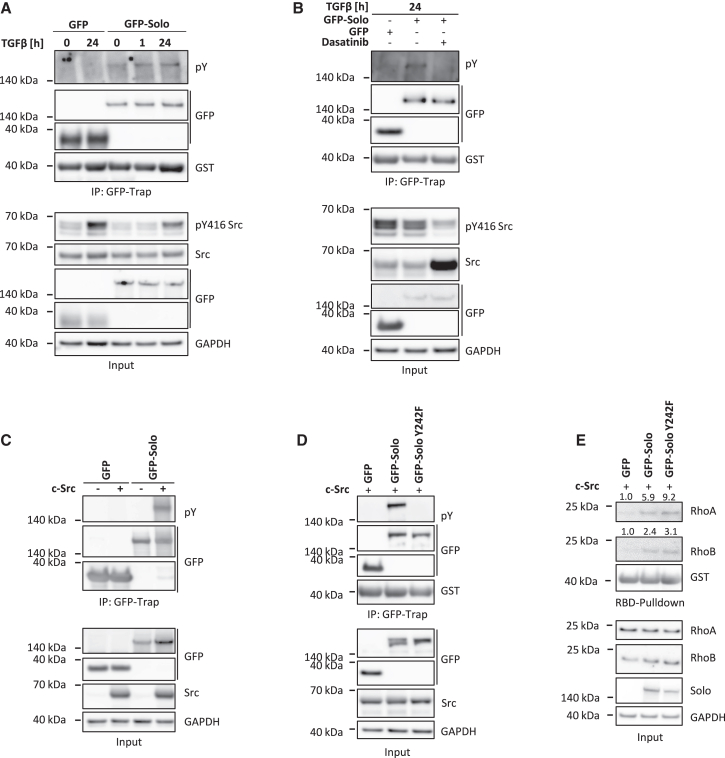
Src phosphorylates Solo at Y242 (A and B) Tyrosine phosphorylation studies in the GFP-Solo MCF10A model system. Cells expressing free GFP were used as control. The cell lines were treated with 10 ng/mL TGFβ for the indicated timepoints and the GFP-tagged proteins were isolated by GFP-Trap. 1 mM Na3VO4 was added to the culture medium 1 h before cell harvesting. (A) The immunoprecipitated material and the total cell lysates were analyzed by immunoblotting with indicated antibodies. GAPDH served as loading control. pY: phosphotyrosine, *n* = 2. (B) The cell lines were treated as in (A), with 20 nM Dasatinib added to one of the GFP-Solo samples 2 h before cell lysis, *n* = 3. (C) Src kinase assay in HEK293T cells. HEK293T cells were transiently transfected with plasmids encoding c-Src (+) or an empty control (−), along with either GFP or GFP-Solo. After 24 h, the cells were lysed and a GFP-Trap assay was performed. The immunoprecipiated material and the total cell lysates were analyzed by immunoblotting with the indicated antibodies. GAPDH served as loading control, *n* = 2. (D) HEK293T cells were co-transfected with plasmids encoding c-Src and GFP, GFP-Solo or GFP-Solo Y242F, respectively. The GFP-tagged proteins were isolated by GFP-Trap 24 h later. The immunoprecipitated material as well as the total cell lysates were analyzed by immunoblotting with the indicated antibodies, *n* = 4. (E) Representative RBD-pulldown assay of HEK293T cells lysed 24 h post transient transfection with the indicated constructs. The immunoprecipitated material as well as the total cell lysates were analyzed by immunoblotting with the indicated antibodies. Densitometric analysis was used to indicate the normalized fold change in active RhoA and RhoB relative to GFP (see also Figure S5). GAPDH served as a loading control, *n* = 3.

RhoGEFs are often regulated by phosphorylation to ensure the accurate spatiotemporal control of Rho GTPase signaling pathways.^15^ To explore the potential regulation of Solo by post-translational modification, we isolated GFP-Solo using GFP-Trap from both TGFβ-treated and untreated MCF10A cells. Database mining revealed tyrosine 242 (Y242) as the most abundantly detected phosphorylation site on Solo, as identified in several global mass spectrometry studies (PhosphoSitePlus^16^). Therefore, we first probed the immunoprecipitated protein with an anti-phosphotyrosine (pY) antibody. Notably, the low levels of GFP-Solo tyrosine phosphorylation under basal conditions were enhanced by the TGFβ treatment (Figure 2A). Given that the non-receptor tyrosine kinase Src is activated by TGFβ^17^ (Figure 2A) and Src contributes to cell adhesion and motility, we investigated if the tyrosine phosphorylation of Solo is dependent on Src. For this, we treated the cells with the Src family kinase (SFK) inhibitor dasatinib for 1 h prior to harvesting for pull-down experiments. The inhibitor strongly reduced active pY416 Src levels and led to a stabilization of Src (Figure 2B), which was also observed in previous studies.^18^^,^^19^ Importantly, Src inhibition fully abolished the GFP-Solo phosphorylation in the TGFβ-treated cells (Figure 2B). For further characterization of the SFK-dependent phosphorylation of Solo we used HEK293T cells, which show a high transfection efficiency. These biochemical experiments confirmed that the phosphorylation of GFP-Solo was readily detected only in cells co-transfected with Src (Figure 2C). Prompted by these results, we examined whether Y242 represents the Src phosphorylation site. Indeed, substituting Y242 with phenylalanine (Y242F) completely eliminated GFP-Solo tyrosine phosphorylation in Src-co-transfected HEK293T cells. (Figure 2D).

Although Y242 is not located within the GEF domain of Solo, this phosphorylation could potentially modulate RhoGEF activity indirectly. To investigate this, RBD-pulldown assays were performed on lysates isolated from HEK293T cells co-expressing Src and the GFP-Solo variants. Cells co-transfected with wild-type GFP-Solo exhibited increased RhoA and RhoB activity compared to those co-transfected with Src and GFP (Figures 2E, S5A, and S5B). This is in line with the well-established RhoA/B GEF activity of Solo previously observed in other cell systems.^10^^,^^11^^,^^12^ By comparison, the GEF activity of the Y242F mutant was elevated relative to wild-type Solo, although this trend was not statistically significant (Figures 2E, S5A, and S5B). Collectively, these experiments identify the RhoGEF Solo as a novel Src substrate.

### Solo phosphorylation by Src on Y242 promotes the motility of MCF10A cells

**Figure 3:**
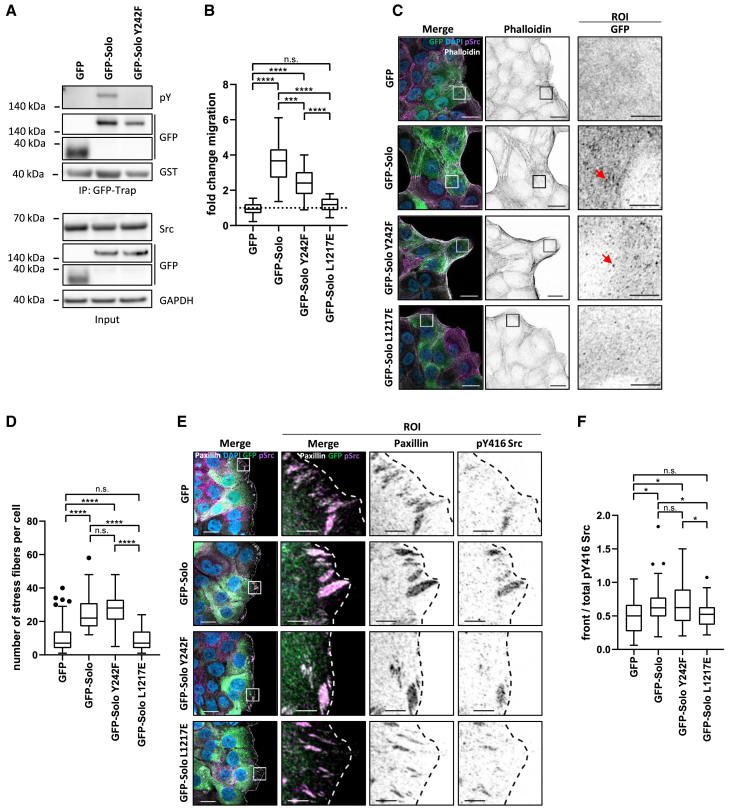
Solo regulates pY416 Src localization in MCF10A cells and its phosphorylation by Src further enhances cell motility (A–D) Validation and phenotypic analysis of the MCF10A cell model with constitutive overexpression of Src and doxycycline inducible expression of GFP-tagged wild-type or Y242F mutated Solo (see also Figure S6). Cells co-expressing Src and free GFP were used as a control. The expression of all inducible constructs was activated by doxycycline addition three days before performing the assays. (A) GFP-Trap assay validating the phosphorylation of Solo at Y242 by Src in the GFP-Solo + Src MCF10A model system. The cells were treated for 1 h with Na3VO4 before lysis and GFP-Trap. The immunoprecipitated material and the total cell lysate were analyzed by immunoblotting with the indicated antibodies. GAPDH served as loading control. pY: phosphotyrosine, *n* = 3. (B) Haptotactic migration assay of the indicated stable cell line. Cells were seeded on Collagen-R coated transwells. 24 h later, the cells were fixed, stained with crystal violet and imaged. The number of cells per field of view was counted in ImageJ, and normalized to the values obtained for the GFP control. The latter was set to 1 and is indicated with the dotted line. Cells co-expressing Src with the GEF inactive Solo L1217E mutant were included as reference. Data shown as Tukey boxplots where each dot represents one field of view. Statistical significance was determined by Brown-Forsythe and Welch ANOVA with Games-Howell’s multiple comparisons test, *n* = 4, *N* = 34–40. (C) Representative fluorescence microscopy images of the cell systems used in (B). The MCF10A cells were plated on Collagen-R coated coverslips and fixed 24 h later. After permeabilization, the cells were immunostained for pY416 Src (pSrc, purple). The actin cytoskeleton was stained with phalloidin (white in the merge and greyscale in the single channel image) while the DNA was counterstained with DAPI (blue). Images show representative single confocal planes, which were acquired and are displayed with identical settings. Scalebar 20 μm. ROIs show a magnification of the regions indicated on the merged image by the white squares; red arrows point to exemplary vesicular structures observed in the GFP channel (see also Figure S7). Scalebar 5 μm. (D) Quantification of the number of stress fibers per cell. Data shown as Tukey boxplots where each dot represents one cell. Statistical significance was determined by one-way ANOVA with Tukey’s multiple comparison, *n* = 3, *N* = 49–74. (E) Representative scratch assays coupled with immunofluorescence for Paxillin and pSrc in the cell systems from (C). 48 h after doxycycline induction, the cells were plated on Collagen-R coated coverslips. 24 h later the confluent cell monolayer was manually scratched, followed by fixation 8 h later and immunostaining for pSrc (purple) and Paxillin (white). Nuclei were counterstained with DAPI (blue). Representative confocal images of the maximum intensity projections of several confocal planes, which were acquired and are displayed with identical settings. Dashed lines indicate the scratch front. Scalebar 20 μm. ROI shows a magnification of the regions indicated by the white squares in the merged images. Scalebar 5 μm. (F) Quantification of the pSrc signal at the scratch front in experiments representatively shown in (E). The front of each cell facing the scratch was quantified by measuring the mean fluorescence intensity (MFI) of the pSrc signal in a 5 μm depth relative to the scratch. The resulting ratio of front pSrc to total pSrc was then calculated. Data shown as Tukey boxplots where each dot represents one cell. Statistical significance was determined by Brown-Forsythe and Welch ANOVA with Games-Howell’s multiple comparisons test, *n* = 3, *N* = 39–49. (B, D, and F) Tukey boxplots range from the 25th to 75th percentile with a center line indicating the median. Whiskers extend 25th and 75th percentile by 1.5x inter-quartile distance, outliers are represented by dots. ∗∗∗∗*p* < 0.0001, ∗∗∗*p* < 0.001, ∗∗*p* < 0.01, ∗*p* < 0.05, n.s. not significant.

To study the cellular consequences of Solo phosphorylation, we established a MCF10A model system with co-expression of Src and the GFP-Solo variants of interest (Figure S6). After confirming the phosphorylation of Solo at Y242 in this model (Figure 3A), we performed transwell migration assays to determine if this post-translational modification impinges on Solo’s positive regulatory role in cell motility. As shown in Figure 3B, cells co-expressing Src and wild-type GFP-Solo exhibited a significant increase in motility compared to the control cells with Src and GFP. This increase was dependent on the RhoGEF activity of Solo. Notably, cells co-expressing Y242F GFP-Solo displayed reduced motility compared to those co-expressing the wild-type protein, although their motility remained significantly higher than that of the GFP control. Together, these experiments indicate that Solo and Src cooperate to drive MCF10A cell motility, with Solo phosphorylation at Y242 by Src playing a critical role in this process.

### Solo regulates Src localization to the leading edge in MCF10A cells

We next sought to characterize the molecular basis of the cooperation between Src and Solo. High-resolution imaging of the stable cell lines revealed that cells co-expressing Src with GFP-Solo or GFP-Solo Y242F exhibited prominent stress fibers and showed a significant increase in F-actin staining compared to cells co-expressing Src with free GFP or GEF-inactive Solo (Figures 3C and S7). Consistently, quantification showed a significant increase in the number of stress fibers per cell for GFP-Solo and GFP-Solo Y242F compared to controls (Figure 3D), a functional indicator of active RhoA.^20^ Although the number of stress fibers was slightly elevated in GFP-Solo Y242F expressing cells no significant difference was observed among WT and Y242F Solo. These results indicate that the Y242F mutant is GEF competent in the MCF10A model system, which is in line with the biochemical data obtained with the HEK293T cells (Figure 2E). Interestingly, image analysis also revealed a vesicular localization of Solo, particularly prominent for the wild-type and the Y242F mutated protein (Figure 3C, ROI). We previously observed Solo on RhoB positive vesicles, and showed that Solo regulates the activity of RhoB on endosomes.^10^ Given that Src is trafficked from the perinuclear region to the cell front in a RhoB-dependent manner and this is important for Src activation,^7^^,^^21^ we hypothesized that Solo could regulate Src trafficking and through this positively impinge on cell motility.

To test this hypothesis, we performed scratch assays to induce polarized Src trafficking to the leading edge of cells where Src is known to be active.^7^^,^^22^ To specifically analyze the subcellular distribution of active Src, we immunostained the cells with an antibody reactive with the Src autophosphorylation site pY416. Indeed, we observed a partial colocalization of pY416 Src with the focal adhesion protein Paxillin near the wound front (Figure 3E). Notably, when quantifying the front versus total pY416 Src distribution, we observed a significant increase at the front in cells expressing GFP-Solo compared to GFP control and GFP-Solo L1217E cells. The GFP-Solo Y242F cells showed a similar distribution pattern to wild-type Solo (Figure 3F). Biochemical pY416 analysis in cell lysates did not reveal changes in total Src phosphorylation, indicating that the observed differences in Src activity are related to its localized distribution to focal adhesions, rather than a global increase in Src phosphorylation (Figure S6).

These findings suggest that Solo is not only a Src substrate but in turn also controls Src trafficking and activation in a RhoGEF-dependent manner. However, in this experimental analysis, the regulation appears to be independent of Solo’s phosphorylation at Y242.

### Solo regulates Src trafficking in MDA-MB-231 cells

**Figure 4:**
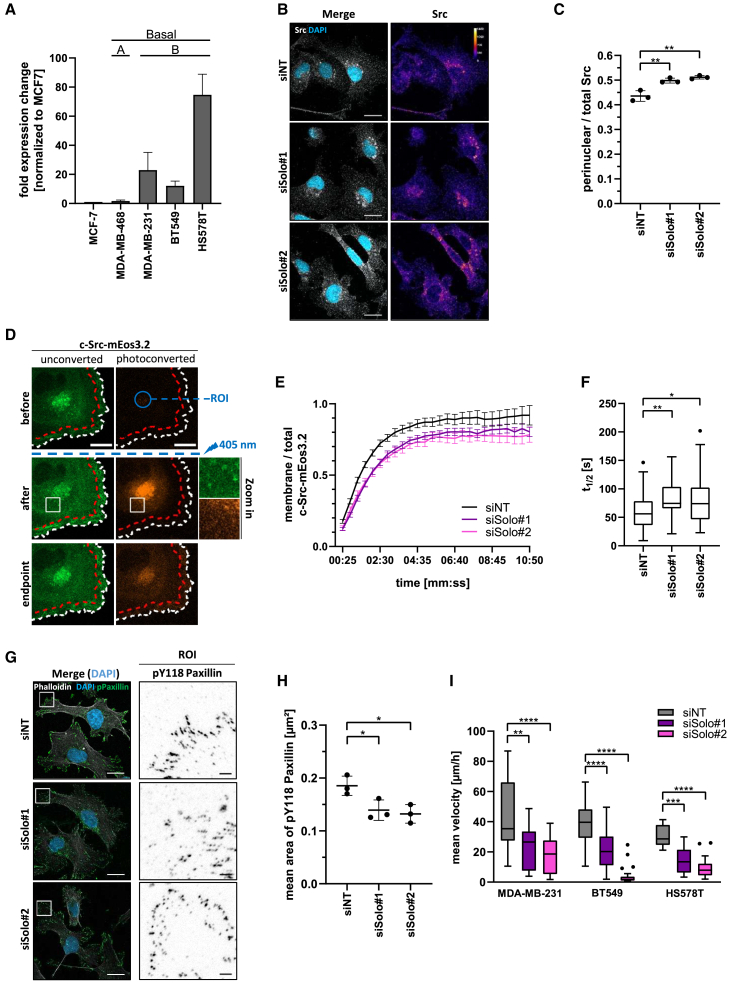
Solo regulates Src trafficking and function in TNBC cell lines (A) Relative qRT-PCR measurements of ARHGEF40/Solo gene expression in a panel of TNBC breast cancer cell lines, normalized to MCF7 levels (see also Figure S8). The molecular classification of the cell lines is annotated on the graph. *n* = 3, shown are means +SD. (B) Representative immunofluorescence stainings and quantification (C) of Src localization in MDA-MB-231 cells with transient Solo depletion. MDA-MB-231 cells were transfected with control (siNT) or two independent Solo specific siRNAs. After 72 h cells were plated on Collagen-R coated coverslips, fixed 4 h later and immunostained for total Src (fire LUT in (B)). The DNA was counterstained with DAPI (blue). Shown are representative confocal images of the maximum intensity projections of several confocal planes, which were acquired and are displayed with identical settings. Scalebar 20 μm. (C) Quantification of perinuclear Src in the images representatively shown in (B). The determination of the perinuclear area was based on the DAPI signal. Shown is mean ± SD, where each dot represents one biological repeat. Statistical significance was determined by one-way ANOVA with Tukey’s multiple comparison, *n* = 3, *N* = 38–40. (D–F) Investigation of Src trafficking in dependence of Solo using the stable c-Src-mEos3.2 MDA-MB-231 cell line (see also Figure S10A). The Src reporter cells were seeded in dishes with a 2-well insert to form a confluent monolayer. After 24 h, the insert was removed, and live cell imaging was performed 6 h later. (D) Validation of the photoconversion parameters. Single cells were imaged before photoconversion, then perinuclear Src was photoconverted by illuminating a fixed-size circular ROI (blue ROI) based on the unconverted signal. Src photoconversion was confirmed immediately after illumination, and the distribution of photoconverted Src was tracked throughout the cell over time until the endpoint. For quantifications shown in (E), for each time point, a cell ROI (total Src, white dashed line) and a membrane ROI (membrane Src, area confined by the red and white dashed lines) were defined based on the cell outline. These ROIs were used to measure the mean fluorescence intensity (MFI). (E) c-Src-mEos3.2 MDA-MB-231 cells were transfected with control (siNT) and two independent Solo specific siRNAs, re-plated 48 h later and used for photoconversion assays as described in (D, see also Figure S10B). The *membrane MFI/cell MFI* ratio of the photoconverted c-Src-mEos3.2 signal is shown over time. 00:00 denotes the timepoint of photoconversion. Shown are means +/− SEM, *n* = 4, *N* = 28–32 cells. (F) t_1/2_ of each cell extracted from fitted curves shown in (E). Data shown as Tukey boxplots where each dot represents one cell. Statistical significance was determined by Brown-Forsythe and Welch ANOVA with Dunett’s T3 multiple comparison, *n* = 4, *N* = 28–32. (G) Representative pY118 Paxillin immunofluorescence images of MDA-MB-231 cells, 72 h post transient depletion of Solo using two independent siRNA cells. siNT treated cells were used as control. pY118 Paxillin is shown in green on the merge and in greyscale in the magnified region of interest (ROI). The actin cytoskeleton was stained with phalloidin (white) and the DNA was counterstained with DAPI (blue). Shown are representative confocal images of the maximum intensity projections of several confocal planes, which were acquired and are displayed with identical settings. Scalebar uncropped image 20 μm; scalebar ROI 5 μm. (H) Mean area of the pY118 Paxillin signal per cell quantified in experiments representatively shown in G, shown is mean ± SD. Statistical significance was determined by one-way ANOVA with Tukey’s multiple comparison, *n* = 3, *N* = 27–28. (I) Quantification of random cell motility of Solo depleted TNBC cell lines (see also Figure S8). Cells were transfected with control (siNT) and Solo specific siRNAs. After 72 h cells were replated on Collagen-R coated substrate and manually tracked for 3 h. Data shown as Tukey boxplots where each dot represents one cell. Statistical significance was determined by one-way ANOVA with Dunnett’s multiple comparison, *n* = 3, *N* = 30–50. (F and I) Tukey boxplots range from the 25th to 75th percentile with a center line indicating the median. Whiskers extend 25th and 75th percentile by 1.5x inter-quartile distance, outliers are represented by dots. ∗∗∗∗*p* < 0.0001, ∗∗∗*p* < 0.001, ∗∗*p* < 0.01, ∗*p* < 0.05, n.s. not significant.

To test the functional relevance of endogenous Solo in the regulation of Src and cell motility in cancer cells, we first analyzed Solo expression in a panel of triple negative breast cancer (TNBC) cells, which often exhibit high levels of EMT TFs as well as SFKs.^23^ In line with the patient sample data (Figure 1A), Solo was found to be highly expressed in several TNBC cell lines, in particular of the basal-like B subtype (Figure 4A), by comparison to the luminal MCF7 cells.

Consequently, we selected the MDA-MB-231 cell line for further experiments. As observed previously for the MCF10A cells, Solo depletion did not alter total Src or pY416 Src levels in cell lysates (Fig S8). While endogenous pY416 Src could not be reliably detected by immunofluorescence staining in MDA-MB-231 cells (data not shown), we did observe an increased perinuclear accumulation of Src in cells with siRNA-mediated Solo depletion (Figures S8, 4B, and 4C). A similar effect was also observed in HS578T cells (Figure S9). These results mirror the effects observed in the MCF10A cell model, suggesting an active role for Solo in the trafficking of Src.

To investigate this hypothesis in a controlled manner, we generated an MDA-MB-231 reporter cell line expressing photoconvertible Src-mEos3.2 based on a previously validated construct of Chen et al.^24^ (Figures S10A and 4D). This system allows for the targeted photoconversion of the perinuclear Src pool and subsequent monitoring of its trafficking and distribution over time (Figure 4D). In scratch assays using control and cells with Solo depletion (Figure S10B), we measured the photoconverted Src MFI of the whole cell and front (white and red regions in Figure 4D, respectively) and calculated the ratio for each time point (Figure 4E). Using the resulting curves, we extracted the t_1/2_ for each cell through a non-linear least squares fitting (Figure 4F). Notably, the median t_1/2_ increased from 55 s for control cells to 75 s for Solo knock down cells. This indicates that Solo modulates the dynamics of Src trafficking from the perinuclear region to the cell periphery in MDA-MB-231 cells.

### Impaired Src trafficking reduces focal adhesion signaling and migration of TNBC cell lines

Based on our findings, we hypothesized that reduced Src trafficking to the cell front would impair phosphorylation of Src substrates at focal adhesions. To test this, we performed immunostaining for Paxillin (Figure S11A) and pY118 Paxillin (Figure 4G) in control and MDA-MB-231 cells with Solo depletion. While the focal adhesion area per cell remained unchanged (Figure S11B), the mean area of pY118 Paxillin decreased from 0.19 μm^2^ to 0.15 μm^2^ upon Solo depletion (Figure 4H). Collectively, these results suggest that Solo loss reduces Src trafficking to the periphery, thereby attenuating Src downstream signaling at focal adhesions.

Src signaling at focal adhesions is critical for focal adhesion turnover and, consequently, efficient cell migration.^25^^,^^26^ To investigate if cell migration is affected in Solo-depleted TNBC cell lines, we performed siRNA-mediated knock down of Solo in MDA-MB-231, BT549, and HS578T cells followed by single cell motility analysis after seeding onto collagen-coated plates (Figure S8). Across all three cell lines Solo knock down significantly decreased the mean velocity per cell (Figure 4I). The medians decreased from 38 to 23 μm/h for MDA-MB-231 cells, and from 40 to 10 μm/h, and 35 to 10 μm/h for BT-549 and HS578T upon Solo knock down, respectively. These data show that the positive contribution of Solo to cell motility is conserved in several cell systems.

## Discussion

Our work identifies the poorly described RhoGEF Solo to be upregulated in the mesenchymal cell state and act as a positive regulator of cell motility in a RhoGEF dependent manner. In line with our findings, Solo was reported to drive directed migration through RhoA signaling in NSCLC cells.^27^ In contrast, in epithelial MDCK cells, Solo was found to decelerate collective but not single cell migration.^28^ Interestingly, the RhoA-targeting PDZ-RhoGEF (PRG) drives cell motility and was recently shown to interact and depend on Solo for its RhoA GEF activity.^29^^,^^30^ Solo’s effect on cell migration may therefore be influenced by the type of migration, the molecular background (epithelial vs. mesenchymal) and abundance of regulating proteins in the specific cell state. As such, we discovered that Src phosphorylates Solo at Y242, which is, to our knowledge, the first characterization of a post-translational modification of Solo. Phosphorylation of RhoGEFs commonly regulates their activity.^15^ For example, Src-induced phosphorylation of GEF-H1 promotes its RhoGEF activity and cell-edge dynamics,^31^ whereas phosphorylation of SGEF reduces RhoGEF activity and cell migration.^32^ Although we did not observe significant global effects on RhoGEF activity for the phosphodeficient Solo Y242F mutant under basal conditions, there was a consistent trend toward increased Rho-GTP levels in the biochemical assay, indicating that phosphorylation might negatively regulate the GEF activity of Solo. Given that the Y242F mutant is less competent at promoting cell motility, a process that integrates dynamic turnover of protein complexes, it is possible that Solo phosphorylation is required for the dynamic regulation of Rho activity cycles. The feedback regulation between Src and Solo may thereby ensure precise spatiotemporal control of Rho GTPase signaling during cell migration, thus enhancing migration efficiency.

Notably, our work has also identified Solo as a key regulator of Src trafficking and activation. While RhoGEF-active Solo promoted targeting of Src to the cell periphery, loss of endogenous Solo reduced Src trafficking dynamics leading to perinuclear Src enrichment. These findings are in line with previous reports showing that correct trafficking and localization of Src relies on the actin cytoskeleton and RhoB.^7^^,^^33^^,^^34^ Altered Src trafficking dynamics and routes can ultimately modulate cellular protrusions and focal adhesion signaling.^35^^,^^36^ Indeed, we found that Solo depletion reduced pY118 Paxillin levels at focal adhesions and dampened cell migration in TNBC cell lines. This could indicate reduced turnover of focal adhesion complexes.^37^ Src trafficking via RhoB-dependent pathways was also reported to involve microtubules in a process regulated by GEF-H1.^38^ Interestingly, our recent work showed that GEF-H1 is highly expressed in TNBC cells, where it contributes to cancer stem cell maintenance.^39^ It is therefore possible that GEF-H1 could to some extent be redundant with Solo in the control of Src trafficking. Future studies could thereby explore the potential crosstalk between Solo and GEF-H1 in the regulation of RhoB-dependent trafficking during cell migration and cellular transformation.

### Limitations of the study

While our work focuses on RhoB, it is possible that Solo’s role in promoting Src trafficking and motility also involves RhoA. In fact, RhoA-dependent rearrangements of the actin cytoskeleton can affect focal adhesion architecture and membrane trafficking in a reciprocal manner. Further studies are thus needed to evaluate Rho GTPase activity dynamics and clarify the interplay between RhoB and RhoA signaling downstream of Solo. While our data demonstrate that Src phosphorylates Solo at Y242, the precise molecular consequences of this modification remain unclear. Solo phosphorylation may facilitate the local recruitment of regulatory partners containing SH2 or PTB domains, which could further contribute to the observed cellular effects. Future mass spectrometry studies could help identify these proteins and further elucidate the molecular mechanisms of the newly discovered Src-Solo signaling node.

## Resource availability

### Lead contact

Further information and requests for resources should be directed to and will be fulfilled by the lead contact, Monilola A. Olayioye (monilola.olayioye@izi.uni-stuttgart.de).

### Materials availability

All unique/stable reagents generated in this study are available from the lead contact with a completed materials transfer agreement.

### Data and code availability

All data reported in this paper will be shared by the lead contact upon request. Any additional information needed to reanalyze the data reported in this paper is available from the lead contact upon request.

## Acknowledgments

We thank Angelika Hausser (University of Stuttgart, Germany) for helpful discussions. We thank Philipp Rathert (University of Stuttgart, Germany) for providing vectors. We acknowledge the support from the Cellular Analytics platform of the Stuttgart Research Center Systems Biology (10.13039/501100009534University of Stuttgart, Germany). The Graphical abstract was created with BioRender.com. This work was supported by the 10.13039/501100001659Deutsche Forschungsgemeinschaft (10.13039/501100001659DFG) grant OL239/11-1 to Monilola A. Olayioye.

## Author contributions

Conceptualization, M.A.O., C.L., and F.M.; methodology: F.M.; software: F.M.; formal analysis: F.M., C.L., R.T., and B.N.; investigation: F.M., C.L., B.N., R.T., D.B., F.F., and M.A.F.; data curation: F.M.; writing – original draft: F.M. and C.L.; writing – review and editing: F.M., C.L., and M.A.O.; visualization: F.M.; supervision: M.A.O., C.L., and F.M.; project administration: M.A.O.; funding acquisition: M.A.O.

## Declaration of interests

The authors declare no competing interests.

## STAR★Methods

### Key resources table

**Table:** 

REAGENT or RESOURCE	SOURCE	IDENTIFIER
**Antibodies**		

Goat anti-GST	Sigma Aldrich	Cat# GE27-4577-01; RRID: AB_771432
Mouse anti-Paxillin	BD Biosciences	Cat# 610052; RRID:AB_397464
Mouse anti-PY20	Santa Cruz Biotechnology	Cat# sc-508; RRID:AB_628122
Mouse anti-PY99	Santa Cruz Biotechnology	Cat# sc-7020; RRID:AB_628123
Mouse anti-RhoA	Santa Cruz Biotechnology	Cat# sc-418; RRID:AB_628218
Rabbit anti-E-Cadherin	Cell Signaling Technology	Cat# 3195; RRID:AB_2291471
Rabbit anti-GAPDH	Sigma Aldrich	Cat# G9545; RRID: AB_796208
Rabbit anti-GFP	Cell Signaling Technology	Cat# 2956S; RRID:AB_1196615
Rabbit anti-N-Cadherin	Cell Signaling Technology	Cat# 13116; RRID:AB_2687616
Rabbit anti-pY118 Paxillin	Cell Signaling Technology	Cat# 2541; RRID:AB_2174466
Rabbit anti-pY416 Src	Cell Signaling Technology	Cat# 6943; RRID:AB_10013641
Rabbit anti-RhoB	Cell Signaling Technology	Cat# 2098; RRID:AB_2179103
Rabbit anti-Solo	Pineda Antibody Service, Berlin, see Lungu et al.^10^	N/A
Rabbit anti-Src	Cell Signaling Technology	Cat# 2109; RRID:AB_2106059

**Bacterial and virus strains**		

*Escherichia coli* DH5α	Thermo Fisher	Cat# 18265017
*Escherichia coli* Stbl3	Thermo Fisher	Cat# C737303

**Chemicals, peptides, and recombinant proteins**		

TGFβ	PeproTech	Cat# 100-21
Dasatinib	Absource Diagnostics	Cat# S1021

**Experimental models: Cell lines**		

BT549	CLS/Cytion	Cat# 300132
HEK293T	ATCC	Cat# CRL-3216
Hs 578T	Bernhard Lüscher, RWTH Aachen University	N/A
LentiX HEK293	Philipp Rathert, University of Stuttgart	N/A
MCF10A cells	Andreas Hecht, University of Freiburg	N/A
MCF10A_EcoR_c-Src_GFP-Solo	This study	N/A
MCF10A_EcoR_GFP-Solo	This study	N/A
MDA-MB-231	CLS/Cytion	Cat# 300275
MDA-MB-231_c-Src-mEos3.2	This study	N/A
Platinum-E	Philipp Rathert, University of Stuttgart	N/A

**Oligonucleotides**		

Primers for cloning: see Table S2		
Hs_ARHGEF40_1_SG QuantiTect Primer Assays (qPCR)	Qiagen	Cat# QT00008589
Hs_PPIA_4_SG QuantiTect Primer Assay (qPCR)	Qiagen	Cat# QT01866137
siNT (ON-TARGETplus® non-targeting control pool)	Dharmacon	Cat# D-001810-10
siSolo#1 (Silencer® Select ARHGEF40)	ThermoFisher Scientific	Cat# s31288
siSolo#2 (ON-TARGETplus Human ARHGEF40)	Dharmacon	Cat# J-030269-12

**Recombinant DNA**		

Gag/Pol	Philipp Rathert, University of Stuttgart	N/A
GST-RBD	Addgene	Cat# 15247
mEos3.2-Lifeact-7	Addgene	Cat# 54696
mOrange2-C-Src-7	Addgene	Cat# 57948
pBabe_c-Src	Addgene	Cat# 26983
pBabe-Hygro	Nancy Hynes	N/A
pCMV	Philipp Rathert, University of Stuttgart	N/A
pCMV_VSV-G	Addgene	Cat#8454
pEGFPC1	Clontech	Cat#6084-1
pEGFPC1-Solo	This study	N/A
pEGFPC1-Solo L1217E	This study	N/A
pEGFPC1-Solo Y242F	This study	N/A
pEGFPC3-HA-Solo	This study	N/A
pGEX6P1-GFP-Nanobody	Addgene	Cat# 61838
pIRESNEO-Scambio-HA	Addgene	Cat# 33354
pLV-EF1a-*c*-Src-mEos3.2-IRES-Puro	This study	N/A
pLV-EF1a-IRES-Puro	Addgene	Cat# 85132
pRK-*c*-Src	Mark Guthridge	N/A
psPAX2	Addgene	Cat# 12260
TRE3G-GFP	Philipp Rathert, University of Stuttgart	N/A
TRE3G-GFP-Solo	This study	N/A
TRE3G-GFP-Solo Y242F	This study	N/A
TRE3G-GFP-SoloL1217E	This study	N/A
TRE3G-MCS-IGPN	Philipp Rathert, University of Stuttgart	N/A

**Software and algorithms**		

Cellpose	https://www.cellpose.org/	
Fiji	https://imagej.net/software/fiji/	
GraphPad Prism 7	GraphPad Software	
ImageQuant TL 8.1	GE Healthcare	
Incucyte® Software	Sartorius	
Python 3.8	https://www.python.org/	
Scansite 4.0	https://scansite4.mit.edu	
Snapgene software	https://www.snapgene.com/	
ZEN Software	Carl Zeiss	

### Experimental model and study participant details

#### Cell culture

MCF10A cells (provided by Andreas Hecht, University of Freiburg) were cultured in DMEM/Ham’s F12 (Gibco) supplemented with 5% horse serum (HS, Gibco), 20 ng/mL epidermal growth factor (EGF, R&D), 500 ng/mL Hydrocortisone (Sigma Aldrich), 10 μg/mL insulin (Sigma Aldrich) and 100 ng/mL cholera toxin (Sigma Aldrich). Stable cell lines were cultured in media supplemented with the corresponding selection antibiotics: Puromycin (Sigma Aldrich, P8833-25mg), G418 (Roth, CP11.3), Hygromycin (Roth, CP12.2). Resuspension medium was DMEM/Ham’s F12 (Gibco) supplemented with 20% horse serum (Gibco). MDA-MB-231, BT549, and HS578T cells were cultured in DMEM (Gibco) supplemented with 5% fetal calf serum (FCS, Gibco). HEK293T and HeLa cells were cultured in RPMI medium (Gibco) with 10% FCS (Gibco). LentiX HEK293 cells (provided by Philipp Rathert, University of Stuttgart) were maintained in DMEM medium (Gibco) with 10% FCS (Gibco), 10 mM HEPES (Thermo Fisher Scientific) and 1 mM Sodium Pyruvate (Thermo Fisher Scientific). All cell lines were grown at 37 °C in a saturated humidity atmosphere containing 5% CO_2_. All cell lines were authenticated, tested negative for mycoplasma (Lonza) and were kept in culture for no longer than 2 months.

For EMT induction, MCF10A cells were treated for 24 h or 7 days with 10 ng/mL TGFβ (PeproTech, 100-21). For the latter, the TGFβ was exchanged every 48 h.

### Method details

#### Plasmids and cloning

The sequence of all primers (indicated in italics) can be found in Table S2. To generate the pEGFPC1-Solo, the coding sequence from Solo was amplified via PCR from pEGFPC3-HA-Solo using the GFP-Solo primer. The insert was subcloned into EcoRI-digested pEGFPC1. The catalytically inactive pEGFPC1-Solo L1217E was generated by QuickChange site directed mutagenesis of pEGFPC1-Solo as a template with the *GFP-Solo L1217E QC* primers. The phospho-deficient pEGFPC1-Solo Y242F was generated by QuickChange site-directed mutagenesis using pEGFPC1-Solo as a template and the *GFP-Solo Y242F QC* primers.

The TRE3G-GFP-Solo plasmids were constructed using Gibson assembly. For this, the TRE3G-MCS-IGPN vector (provided by Philipp Rathert, University of Stuttgart) was digested with XhoI and PacI, while the coding sequence for EGFPC1-Solo was amplified via PCR from the plasmids mentioned above using the *Solo TRE3G* primers.

The pLV-EF1a-*c*-Src-mEos3.2-IRES-Puro was generated by restriction digestion followed by ligation. To this end, the pLV-EF1a-IRES-Puro (a gift from Tobias Meyer; Addgene plasmid #85132) was linearized with EcoRI. The Eos3.2 sequence was amplified from mEos3.2-Lifeact-7 (a gift from Michael Davidson; Addgene plasmid #54696) by PCR using the *mEos3.2 amp* primer and was used to replace the mOrange2 tag in the mOrange2-*c*-Src-7 plasmid (a gift from Michael Davidson; Addgene plasmid #57948). For this, the amplified insert and mOrange2-*c*-Src-7 were EcoRI and AgeI digested, and subcloned in the linearized pLV-EF1a-IRES-Puro. TRE3G-MCS-IGPN and TRE3G-eGFP were gifts from Philipp Rathert (University of Stuttgart). pBabe was a gift from Nancy Hynes (Friedrich Miescher Institute for Biomedical Research). pBabe_*c*-Src was a gift from Joan Massague (Addgene plasmid #26983,^40^). pRK-*c*-Src was a gift from Mark Guthridge (Deakin University).

The identity of all constructs was confirmed by Sanger sequencing (Microsynth Seqlab, Göttingen, Germany).

#### Generation of MCF10A_EcoR_GFP-Solo and MCF10A_EcoR_c-Src_GFP-Solo cell line

To generate the MCF10A_EcoR_GFP-Solo cell lines (referred to as GFP-Solo MCF10A), MCF10A_EcoR cells^39^ were transduced with ecotropically packaged retrovirus containing either TRE3G-GFP or one of the TRE3G-GFP-Solo variants. To this end, Platinum-E cells were transfected with 20 μg vector and 10 μg pCMV-Gag/Pol helper vector (a gift from Philipp Rathert, University of Stuttgart) and the supernatant containing retrovirus was filtered and added dropwise to the MCF10A_EcoR cells. Transduced cells were selected with 750 μg/mL G418 and 1.5 μg/mL puromycin, followed by a 1-day doxycycline induction (1 μg/mL) and FACS enrichment (BD Biosciences, FACSAria III). After enrichment, the cells pools were maintained in the absence of doxycycline and validated by immunofluorescence and western blotting.

To generate the MCF10A_EcoR_c-Src_GFP-Solo cell line (referred to as Src-Solo MCF10A), MCF10A_EcoR cells were transduced with ecotropically packaged retrovirus containing either pBabe, or pBabe_c-Src as described above. Transduced cells were selected with 250 μg/mL Hygromycin and 1.5 μg/mL puromycin and validated by immunofluorescence and western blotting. In a second step, MCF10A_EcoR_c-Src cells were transduced with ecotropically packaged retrovirus containing either TRE3G-GFP or one of the GFP-Solo variants and selected with 150 μg/mL hygromycin, 1.5 μg/mL puromycin, and 750 μg/mL G418 for 5 days. Cells pools were maintained in the absence of doxycycline and validated by immunofluorescence and western blotting.

#### Generation of the MDA-MB-231_c-Src-mEos3.2 reporter cell line

Lentiviral particles were produced using LentiX HEK293T cells (a gift from Philipp Rathert, University of Stuttgart), which were transfected with pLV-EF1a-*c*-Src-mEos3.2-IRES-Puro and the packaging plasmid psPAX2 (a gift from Didier Trono, Addgene #12260) and pCMV_VSV-G (a gift from Bob Weinberg, Addgene #8454,^41^). The filtered supernatant was diluted 1:4 with growth media and added to the MDA-MB-231 cells. Transduced cells were selected with 1.5 μg/mL puromycin and validated by immunofluorescence and western blotting.

#### Cell lysis, SDS-PAGE and western blotting

After a washing step with ice-cold PBS, the cells were lysed for 20 min on ice with lysis buffer (150 mM Tris-HCl pH 7.5, 500 mM NaCl, 1% (v/v) Triton X-100, 0.5% (v/v) sodium deoxycholate, 0.1% (w/v) SDS, 10% Glycerol (v/v), 10 mM MgCl_2_, 1 mM DTT, 1 mM sodium orthovanadate, 10 mM sodium fluoride, 0.5 mM PMSF, 20 mM β-glycerophosphate and cOmplete™, EDTA-free Protease Inhibitor Cocktail [Roche]). The cleared lysate was obtained by a 10 min centrifugation at 16.000*g*, 4°C, the protein concentration was determined using the DC Protein Assay (Bio Rad, 5000111) according to the manufacturer instructions. Equal protein amounts were separated by SDS-PAGE (NuPage® Novex® 4%–12% Bis-Tris gels, Invitrogen) and transferred to nitrocellulose membranes using an iBlot® device (iBlot®Gel Transfer Stacks; Invitrogen). The membranes were blocked for at least 1 h at RT with 0.5% (v/v) blocking reagent (Roche) in PBS containing 0.05% (v/v) Tween 20 and 0.01% (v/v) Thimerosal and then incubated with primary antibodies, overnight at 4°C, followed by 1 h incubation with HRP-conjugated secondary antibodies at RT. All antibodies were diluted in blocking solution. The chemiluminescence signal was detected using an Amersham™ Imager 600 or 800 device (GE Healthcare) followed by quantification of the 16-bit images in the linear range using the inbuilt ImageQuant TL 8.1 software. For SFK inhibition, cells were treated with 500 nM Dasatinib (S1021, Absource Diagnostics) for 1 h before lysis.

#### RBD-pulldown

The GST-RBD protein was produced and isolated as described before.^10^ Transfected HEK293T cells were washed on ice once with ice-cold TBS (50 mM Tris-HCl pH 7.6, 140 mM NaCl) and lysed in ice-cold RBD-lysis buffer (150 mM Tris-HCl pH 7.5, 500 mM NaCl, 1% (v/v) Triton X-100, 0.1% (w/v) SDS, 0.5% (v/v) sodium deoxycholate, 1 mM DTT, 10% (v/v) Glycerol, 10 mM MgCl_2_, 1 mM EGTA, 1 mM sodium orthovanadate, 10 mM sodium fluoride, 0.5 mM PMSF, 20 mM β-glycerophosphate and cOmplete™, EDTA-free Protease Inhibitor Cocktail [Roche]). After passing the lysates 5x through a 27G-needle and centrifugation for 5 min at 16.000 g, 4°C, 10% of the cleared lysates was set aside as total cell lysates. Equal volumes of the remaining lysates were incubated with 30 μg GST-RBD protein pre-bound to glutathione Sepharose beads (17-5132-01, Cytiva) for 45 min, 4°C, on an end-over-end rotator with 12 rpm. GST-RBD-beads were washed 4 times with RBD-lysis buffer and 1 time with RBD-salt-exchange buffer (150 mM Tris-HCl pH 7.5, 150 mM NaCl, 1 mM DTT, 10% (v/v) Glycerol, 10 mM MgCl_2_, 1 mM EGTA, 1 mM sodium orthovanadate, 10 mM sodium fluoride, 0.5 mM PMSF, 20 mM β-glycerophosphate and cOmplete™, EDTA-free Protease Inhibitor Cocktail [Roche]). The bound proteins were eluted with 15 μl 4x LDS (NP0008, Invitrogen), and subjected to SDS-PAGE and western blotting as described above. All total cell lysates and pulldown samples from one experiment were run on the same gel/blot and the pulldown-total cell lysate densitometric ratios were normalized to control cells.

#### GFP-trap

The GFP trap beads were prepared in house according to the protocol described in Katoh et al.^42^ For each pulldown reaction, 18 ng GST-coupled GFP-Nanobody (a gift from Kazuhisa Nakayama; Addgene plasmid #61838) were incubated with Glutathione Sepharose beads (GE17-5132-01, Sigma) for 1 h in GFP-trap buffer (50 mM Tris-HCl pH 7.5, 10% (v/v) Glycerol, 150 mM NaCl, 0.5 mM PMSF, 1% (v/v) Triton X-100, 1 mM sodium orthovanadate, 10 mM sodium fluoride, 20 mM β-glycerophosphate and cOmplete™, EDTA-free Protease Inhibitor Cocktail [Roche]) with end-over-end rotation at 4°C. The beads were washed twice with ice-cold GFP-Trap buffer before being used for the pulldown.

At 24 h post transient transfection, HEK293 cells were treated with 1 mM sodium orthovanadate for 1 h. The cells were then lysed on ice for 15 min using ice-cold GFP-Trap buffer. The full cell lysate was subsequently centrifuged for 10 min at 16.000 g and 4°C. 10% of the cleared lysate were saved as input sample and 90% used for the GFP-trap, whereby the pre-cleared lysate was incubated with the GFP-Trap beads for 2 h at 4°C with end-over-end rotation. To remove unspecific bound protein, the beads were washed once with GFP-Trap buffer, 2 times with high-salt buffer (same as GFP-trap buffer but with 500 mM NaCl), and once with GFP-Trap buffer. Bound proteins were eluted with 15 μl 4x LDS (NP0008, Invitrogen) and subjected to SDS-PAGE and western blotting as described above.

#### RNA interference and plasmid transfection

For siRNA mediated knock downs, Lipofectamine® RNAiMAX (Invitrogen, 13778-150) was used according to the manufacturer. Cells were used for follow up analysis at 72 h after reverse transfection. The following siRNAs were used: siSolo#1 (Silencer® Select ARHGEF40 s31288 from ThermoFisher Scientific) and siSolo#2 (ON-TARGETplus Human ARHGEF40 siRNA J-030269-12 from Dharmacon). Negative control siNT (ON-TARGETplus® non-targeting control pool D-001810-10 from Dharmacon). For plasmid transfection of HEK293T cells PEI 25K™ (23966-100, Polysciences) was used using a 1:3 DNA:PEI ratio. Experiments continued after 24 h.

#### Antibodies

The following primary antibodies were used: anti-RhoA (26C4 SC418, WB: 1:1000), anti-PY99 (sc-7020, WB: 1:500) and anti-PY20 (sc-508, WB: 1:500) from Santa Cruz Biotechnology. Anti-pY416 Src (6943, WB: 1:500, IF: 1:250), anti-Src (2109S, WB: 1:500, IF: 1:250), anti-GFP (2956S, WB: 1:2000), anti-E-Cadherin (#3195, WB: 1:500), anti-N-Cadherin (#13116, WB: 1:500), anti-pY118 Paxillin (#2541, IF 1:200), anti-RhoB (2098S, WB: 1:500) from Cell Signaling Technology. anti-GAPDH (G9545, WB: 1:5000), and anti-GST (GE27-4577-01, WB: 1:1000) from Sigma Aldrich. Anti-Paxillin (610052, WB: 1:1000, IF: 1:200) from BD. The Anti-Solo was custom made (Pineda Antibody Service, Berlin; see^10^).

The following secondary antibodies were used: HRP-labelled secondary goat α-mouse-, goat α-rabbit-, and donkey α-goat-IgG from Dianova (115-035-062, 111-035-144, 705-035-147; WB: 1:10.000). Alexa-Fluor®-labelled secondary IgGs (see Reagents Tools Table), Alexa-Fluor®-633-labelled phalloidin (A22284, IF: 1:2.500) from Invitrogen. DAPI (D8417-5MG, IF: 1:5000) from Sigma-Aldrich.

#### Immunofluorescence

Glass coverslips were coated either ON at 4°C or 2 h at 37°C with 25 μg/mL Collagen-R (Serva). After 2 washing steps with PBS, the cells were seeded in either full media (10% serum) or under reduced serum (0.5% serum) conditions. For fixation, the cells were washed once with prewarmed PBS supplemented with Ca^2+^ and Mg^2+^ (PBS++, Gibco) and incubated 15 min with 4% (w/v) Paraformaldehyd (PFA). After 3 washing steps with PBS++, the PFA was quenched with 150 mM Glycine in PBS for 15 min. Cells were permeabilized with 0.1% (v/v) Triton in PBS++ followed by 3 washing steps with PBS++ and 1 h incubation in blocking solution (PBS++ containing 5% (v/v) goat serum (Invitrogen) and 0.1% (v/v) Tween 20). Alternatively, to stain focal adhesion proteins, cells were fixed and stained concurrently using 4% (w/v) PFA and 0.1% (v/v) Triton X-100 in PBS for 10 min before quenching and blocking. Primary antibody-dilutions were prepared in blocking solution and incubated ON at 4°C in a humidity box. After 3 washing steps with PBS++, the coverslips were incubated with secondary antibodies, DAPI, and/or Phalloidin diluted in blocking solution and incubated for 1 h at RT. Coverslips were washed 3 times with PBS++ before being mounted with ProLong™ Gold Antifade mountant (Molecular Probes™, ThermoFisher Scientific) and left for curing ON at RT.

#### Scratch assays

75.000 cells/cm^2^ Src-Solo MCF10A cells were seeded on Collagen-R coated coverslips and expression of GFP-Solo constructs induced with doxycycline. After 48 h the monolayer was manually scratched, washed 2 times with PBS++, and fresh media supplemented with doxycycline added. After 8 h the cells were fixed and stained as described above.

#### Imaging

All samples were analyzed at room temperature using the confocal laser scanning microscope LSM 980 Airyscan 2 (Carl Zeiss) equipped with a Plan Apochromat 63×/1.40 DIC M27 (Carl Zeiss) oil-immersion objective. The following excitation wavelengths and detection window were used: 405 nm and 425–488 nm for DAPI; 488 nm and 495–550 nm for GFP, unconverted mEos3.2, and Alexa-Fluor®-488 coupled probes; 561 nm and 573–621 nm for photoconverted mEos3.2 and Alexa-Fluor®-546 coupled probes; 633 nm and 655–720 nm for Alexa-Fluor®-633 coupled probes. Linear adjustments to brightness, contrast and maximum intensity projections for figures were made using the ZEN software (Carl Zeiss).

#### Live cell imaging c-Src-mEos3.2 cells

2 days after siRNA transfection 7.500 c-Src-mEos3.2 cells (75 μl of 100.000 cells/ml dilution) were replated in both wells of a 35 mm μ-dishes with 2 well insert (one dish per siRNA; 81176, ibidi) and 1.5 mL media added to the surrounding area. The following day, the inserts were removed at staggered times to trigger the scratch stimulus, ensuring that all conditions were imaged 6 h after stimulation. This intentional delay ensured consistent imaging times across all conditions. Cells showing a polarized phenotype were manually selected and imaged in the green and red channel for 30 timepoints, 25 s time interval, and an orthogonal stack of 7 slices. After timepoint 5 the mEos3.2 was photoconverted by illuminating a manually selected fixed-size circular ROI with the 405 nm laser. For this, the following previously evaluated laser settings were used: 20% laser power for 8 iterations with a scan speed of 8.

#### Image analysis

Image analysis was performed in Fiji on the raw CZI files. CZI files were imported in FIJI using Bio-Formats.^43^ All following calculations were done in Python using pandas. Cellpose human-in-the-loop training based on the cyto2 or cyto3 model^44^^,^^45^^,^^46^ was used to obtain *cell masks* and distinguish between individual cells. The Mean fluorescence intensities (MFI) were measured on the sum slices projections. Different approaches were used to analyze the images.

1. To quantify the F-Actin staining, the MFI of phalloidin per *cell mask* was measured and the values obtained for the GFP-Solo expressing cells were normalized to those of the GFP- control.
2. Measurement of the pY416 Src localization in the scratch assays was performed in three steps. First, as the cells were in a confluent monolayer, the leading edge was identified as the boundary between the cells and scratch area. Second, the leading edge was shifted 5 μm inward, toward the cells, to establish the rear boundary of the cell front. The area between the leading edge and this rear boundary was defined as the *cell front*. Finally, the MFIs of pY416 Src were measured both in the entire cell mask and in the defined *cell front* region and the *cell front* MFI/cell mask MFI calculated.
3. Focal adhesion size and signaling activity, as assessed by Paxillin and pY118 Paxillin staining, was based on.^47^ Briefly, for each cell mask, the raw images were pre-processed with background subtraction, contrast limited adaptive histogram equalization (CLAHE) and Gaussian blur. Afterward, a fixed threshold was set and the size of individual focal adhesions measured. The average size per cell per condition was calculated.
4. The perinuclear enrichment of Src was quantified using two ROIs: The cell mask was used to measure the MFI of the whole cell. Next, the nucleus was detected based on the pre-processed and thresholded DAPI signal and enlarged by 5 μm to include the *perinuclear region*. Additionally, the AND operation of the cell mask and the *perinuclear region* ensured that the *perinuclear region* was restricted to the actual cell. The MFIs were measured and the perinuclear region MFI/cell mask MFI per cell per condition was calculated.
5. The quantification of the Src trafficking in the MDA-MB-231 c-Src-mEos3.2 reporter cell line was carried out in three steps. First, the ROIs were detected and the MFIs measured for each individual time point. For this, the before conversion (green) signal was pre-processed and thresholded to define the *cell ROI*. Next, the borders of the leading edge were manually set and the cell ROI was shrunk to define the *membrane ROI*. The cell- and *membrane ROI* were used to measure the cell and membrane MFI of the photoconverted red channel, respectively. Second, the signals were normalized to the background signal. Since the photoconversion occured after time point 5, the first five frames (t_1_–t_5_) were used to calculate background signals by averaging the MFI before photoconversion. After photoconversion (t > 5), the background-corrected membrane MFI/cell MFI was calculated for each time point. A non-linear least squares fitting on $a\times \left(1-\exp\left(-b\times t_{x}\right)\right)$ performed using scipy. From this, the t_1/2_ for each individual cell was extracted.
6. Stress fibers in the maximum intensity projections were annotated manually and counted per cell.

#### Transwells and random motility assays

Cells were harvested and washed 2 times with serum free medium. For haptotaxis assays, the underside of the transwells (8 μm Pore Size, 3422, Costar) was coated with 25 μg/mL Collagen-R (Serva) ON at 4°C. For invasion assays, the upper side of the membrane was coated with 0.39 μg/mL Matrigel™ (BD) in medium supplemented with 0.5% FCS and incubated for 30 min at 37°C to allow polymerization. 17.500 HeLa cells, 50.000 Src-Solo MCF10A cells or 15.000 MDA-MB-231, HS578T, or BT549 cells were seeded in the upper transwell compartment. The media in both compartments was supplemented with 0.5% serum. At 24 h (Src-Solo MCF10A, HeLa) or 6 h (all other cell lines) post seeding, the transwells were fixed with ROTI®Histofix (P087.3, Roth) for 15 min and the cells were stained with 0.1% Crystal violet (v/v). From each transwell 7 different field of views were imaged and the average number of cells per image calculated using Fiji. All transwells experiments included two technical repeats. The values obtained for the perturbation conditions i.e., Solo overexpression or Solo depletion were normalized to the corresponding control cells, as described in the figure legends.

For TGFβ-treated MCF10A cells, the cells were treated with 5 ng/mL TGFβ for 7 days before reverse transfection with the indicated siRNAs. 72 h post transfection the cells were harvested in serum-free medium and subjected to transwell migration (6 h) and invasion assays (24 h) toward media supplemented with 10% FCS. The values obtained for the Solo depleted cells were normalized to those of the TGFβ-treated siNT cells.

For random motility, 96-well plates were coated with 25 μg/mL Collagen-R (Serva) ON at 4°C. 500 cells resuspended in media containing 0.5% FCS were seeded in duplicate per well for each condition. Images were taken every 10 min using the Incucyte® S2 (Sartorius) for up to 24 h. For quantification, the raw images were exported using the Incucyte® Software and image stacks were generated in FIJI. Focus stabilization was performed using the FIJI plugin Template Matching (Qingzong Tseng, https://sites.google.com/site/qingzongtseng). The cells were manually tracked at the interval 3 h–9 h after attachment using TrackMate^48^ and a python pipeline used to calculate the average speed per cell.

#### ARHGEF40/Solo TCGA BRCA gene expression analysis

Target gene expression data was retrieved from The Cancer Genome Atlas Breast Cancer (TCGA-BRCA, TCGA Research Network: https://www.cancer.gov/tcga) cohort RNAseq dataset. *ARHGEF40* gene (encoding for SOLO) mRNA expression levels were compared between high (*n* = 93) versus low (*n* = 74) EMT signature groups, visualized as log2[*ARHGEF40* expression (FPKM) + 0.01]. The EMT signature was defined by the intersection of the sample groups representing either the highest or the lowest expression quartiles (quantile >0.25 or <0.25) of the four EMT markers *SLUG*, *ZEB1*, *ZEB2* and *TWIST2*. Statistical analysis was done using a two-tailed unpaired t-test with Welch’s correction in GraphPad Prism 9.

#### Quantitative real-time PCR

RNA was isolated using the NucleoSpin® RNA Kit (Macherey-Nagel) according to the manufacturer. 100 ng RNA were used with the Power SYBR® Green RNA-to-CT™ 1-step kit (applied Biosystems) for real-time PCR in the CFX96 Touch Real-Time PCR Detection System (Bio-RAD, 1855196). Hs_ARHGEF40_1_SG QuantiTect Primer Assays (Qiagen) was used to detect ARHGEF40/Solo transcripts. The values were normalized to the housekeeping genes GAPDH (5’-CCCCTTCATTGACCTCAACTA-3’; 5’-CGCTCCTGGAAGATGGTGAT-3’) or PPIA (Hs_PPIA_4_SG QuantiTect Primer Assay, Qiagen) and changes in gene expression levels determined using the 2^−ΔΔCt^-method.

### Quantification and statistical analysis

Data were analyzed and plotted using GraphPad Prism 7. Information about the statistical tests used as well as about sample size N and number of independent repeats can be found in the corresponding figures. Detailed information about the *p*-values can be found in Table S1.

## Supplemental information

Document S1. Figures S1–S11 and Tables S1 and S2

Document S2. Uncropped western blots
