# Supplementary material for: Reciprocal regulation of Solo and Src orchestrates Src trafficking to promote mesenchymal cell migration
Source: iScience. 2025 May 9;28(6):112618. doi: 10.1016/j.isci.2025.112618 (PMC12152665; doi:10.1016/j.isci.2025.112618)
Supplement: Document S1. Figures S1–S11 and Tables S1 and S2 [file mmc1.pdf]

iScience, Volume *28*

Supplemental information

Reciprocal regulation of Solo and Src orchestrates Src trafficking to promote mesenchymal cell migration

Florian Meyer, Cristiana Lungu, Bettina Noll, David Benz, Felix Fränkle, Miguel Â. Ferreira, Raluca Tamas, and Monilola A. Olayioye

Supplemental Information

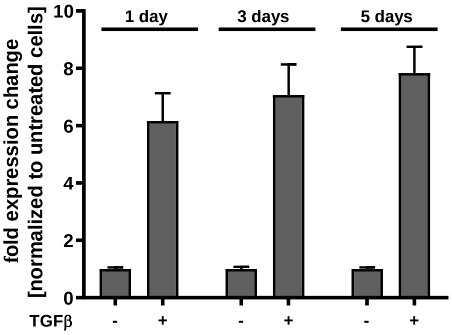

Figure S1: Solo transcript levels are upregulated upon TGFβ treatment of MCF10A cells, related to Figure 1

Relative qRT-PCR measurements of ARHGEF40/Solo gene expression in MCF10A cells treated with 5 ng/ml TGFβ for the indicated time points. Data was normalized to the corresponding untreated cells. Shown are means + SD, n=1.

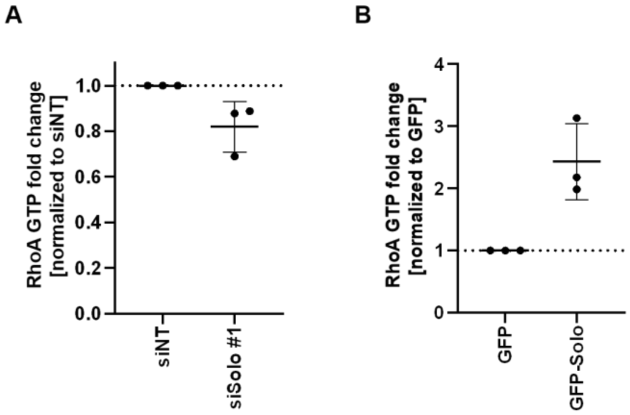

Figure S2: Solo regulates RhoA GTP levels in MCF10A cells, related to Figure 1

Quantification of RBD-pulldown assay in MCF10A transfected with control (siNT) or Solo specific siRNA (**A**), or stable GFP-Solo MCF10A cells (**B**). The RhoA signal obtained for the knock down (A) and GFP-Solo expressing cells (B) was normalized to the values of the respective control. The latter was set to 1 and indicated with a dotted line. Each dot stands for one biological repeat. Line indicates mean +/- SD. n=3.

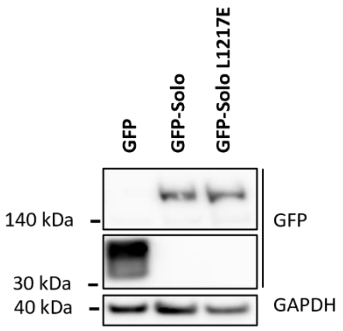

Figure S3: Validation of the GFP-Solo MCF10A model system, related to Figure 1

MCF10A cell lines expressing inducible GFP, wild-type, or catalytically inactive GFP-Solo L1217E were generated by retroviral transduction. 72 h after doxycycline addition, cell lysates were prepared and analyzed by immunoblotting with an anti-GFP antibody. GAPDH served as a loading control, n=3.

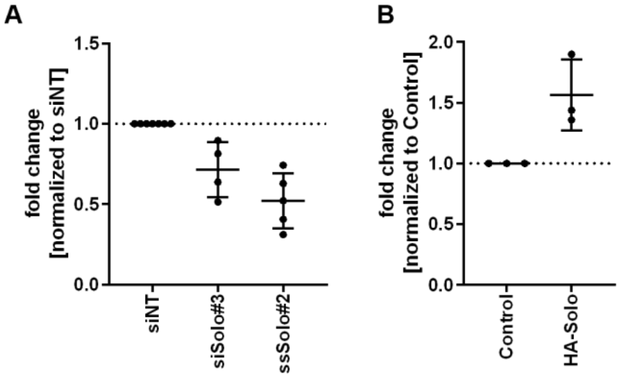

Figure S4: Solo regulates directed cell motility in HeLa cells, related to Figure 1

HeLa cells were transfected with control (siNT) or two independent Solo specific siRNAs (**A**), or control vector and a vector encoding HA-tagged Solo (**B**). After 72 h (A) or 24 h (B), cells were seeded onto matrigel-coated transwell chambers, with the underside coated with collagen, and allowed to invade overnight toward a serum gradient. The values were normalized to the values of the respective control. The latter was set to 1 and indicated with a dotted line. Shown is mean +/- SD, where each dot represents one biological repeat. n=3

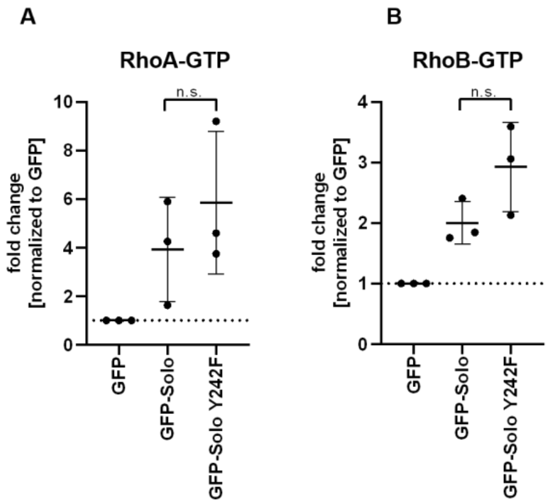

Figure S5: The phosphodeficient Solo Y242F mutant shows a trend toward enhanced RhoGEF activity, related to Figure 2

Quantification of RBD-pulldown assay representatively shown in Fig 2E. (**A**) The RhoA and (**B**) RhoB signal obtained for the GFP-Solo and the GFP-Solo Y242F transfected cells was normalized to the values of the GFP control. The latter was set to 1 and indicated with the dotted line. Each dot stands for one biological repeat. Line indicates mean +/- SD. Statistical significance was determined by unpaired two-tailed t-test, n=3, n.s. not significant.

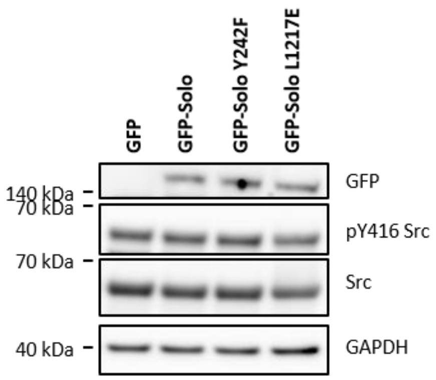

Figure S6: Validation of the MCF10A model systems with co-expression of Src and GFP-Solo variants, related to Figure 3

Stable MCF10A cell lines with constitutive expression of Src and doxycycline inducible expression of the indicated GFP-tagged constructs were generated by retroviral co-transduction. Doxycycline was supplemented to the cell culture medium 72 h before total cell lysate isolation. The samples were analyzed by immunoblotting with the indicated antibodies. GAPDH served as a loading control, n=2.

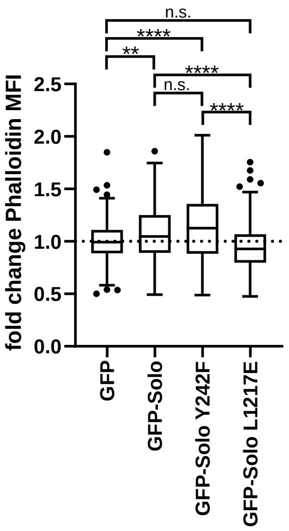

Figure S7: Solo expression increases actin MFI, related to Figure 3

Single cell quantification of the mean fluorescence intensity (MFI) of phalloidin in the images representatively shown in (Fig. 3C) and normalized to the values obtained for the GFP expressing cells. The latter was set to 1 and indicated with the dotted line. Data shown as Tukey boxplots where each dot represents one cell. Statistical significance was determined by Brown-Forsythe and Welch ANOVA with Games-Howell's multiple comparisons test, n=3, N=186-213.

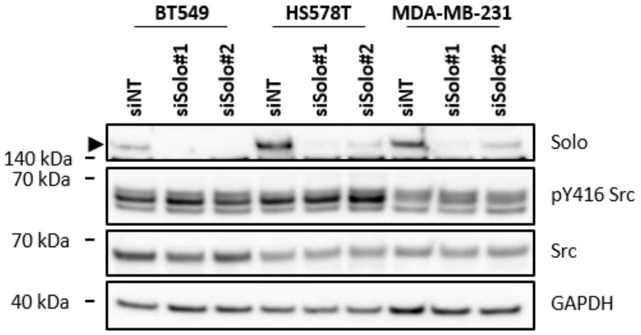

Figure S8: Immunoblot analysis of TNBC cell line panel with Solo depletion, related to Figure 4

The indicated cell lines were transfected with control (siNT) and two independent Solo specific siRNAs. After 72 h, the cells were lysed and protein levels were analyzed by immunoblotting with the indicated antibodies. The arrow indicates the expected molecular weight for endogenous Solo, n=1.

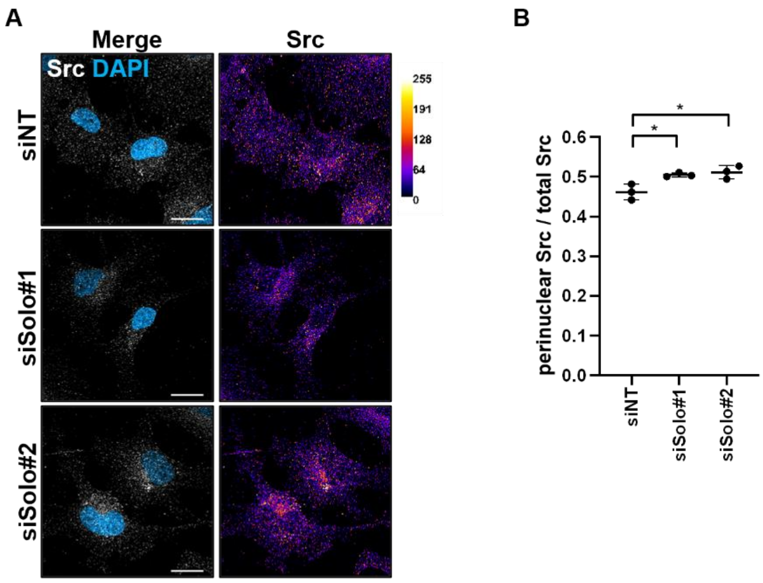

Figure S9: Solo regulates Src localization in HS578T cells, related to Figure 4

(**A, B**) HS578T cells were transfected with control (siNT) or two independent Solo specific siRNAs. After 72 h cells were plated on Collagen-R coated coverslips, fixed 4 h later and immunostained for total Src (fire LUT in (A), low to high intensity displayed from dark purple to white, respectively). The DNA was counterstained with DAPI (blue). Shown are representative confocal images of the maximum intensity projections of several confocal planes, which were acquired and are displayed with identical settings. Scalebar 20 µm. (**B**) Quantification of perinuclear Src in the images representatively shown in (**A**). The determination of the perinuclear area was based on the DAPI signal. Shown is mean +/- SD, where each dot represents one biological repeat. Statistical significance was determined by one-way ANOVA with Tukey's multiple comparison, n=3, N=40-46.

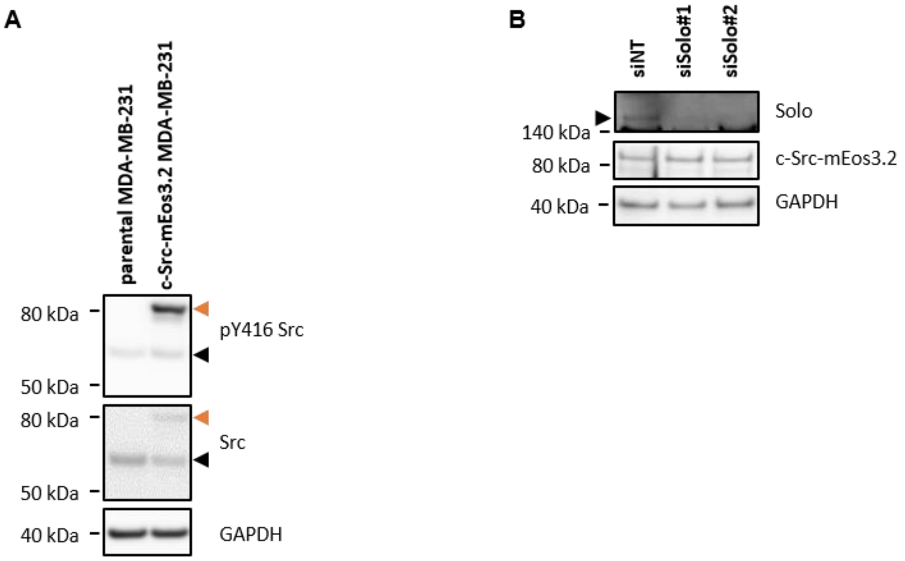

Figure S10: Validation of the c-Src-mEos3.2 MDA-MB-231 cell line, related to Figure 4

(**A**) Expression of c-Src-mEos3.2 in stable MDA-MB-231 cell line. Parental MDA-MB-231 cells and reporter cells stably transduced with c-Src-mEos3.2 were lysed and the samples were subjected to immunoblotting with the indicated antibodies. Orange and black arrows indicate expected molecular weights for the overexpressed mEos3.2-tagged c-Src and endogenous Src, respectively, n=1. (**B**) Solo knock down validation in the c-Src-mEos3.2 MDA-MB-231 cell line. Cells were transfected with either control siRNA (siNT) or two independent siRNAs targeting Solo. After 72 h the cells were lysed and the immunoblotted samples were probed with the indicated antibodies. The arrow indicates the expected molecular weight for endogenous Solo, n=1.

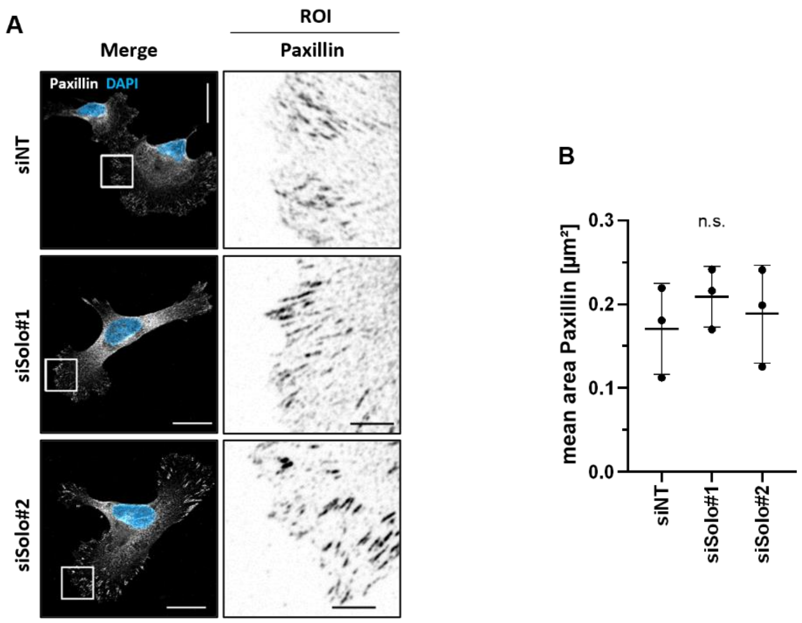

Figure S11: Solo depletion in MDA-MB-231 cells does not affect focal adhesion area, related to Figure 4

(**A**) Quantification of mean focal adhesion area per cell as based on the Paxillin immunostaining of MDA-MB-231 cells with Solo depletion. The cells were transfected with control (siNT) and two independent Solo specific siRNAs. After 72 h, the cells were replated on Collagen-R coated coverslips and fixed 4 h later. Paxillin immunostaining (white) was used to identify focal adhesions, the DNA counterstained with DAPI (blue). Shown are representative maximum intensity projections of several confocal planes, which were acquired and are displayed with identical settings. Scalebar of uncropped image 20 µm; ROI 5 µm. (**B**) Quantification of mean Paxillin area in experiments representatively shown in (A). Each dot stands for one biological repeat. Line indicates mean +/- SD, Statistical significance was determined by one-way ANOVA with Tukey‘s multiple comparisons, n=3, N=25-27, n.s. not significant.

Table S1: p-Value Summary, related to STAR methods.

Figure		Samples	n	p value	p value summary
**Figure 1**	A	high (n = 74) vs. low (n = 93)		< 0.0001	****
	D	siNT + TGFb vs. siNT	3	< 0.0001	****
		siNT + TGFb vs. siSolo#1	3	< 0.0001	****
		siNT + TGFb vs. siSolo#2	3	< 0.0001	****
	E	siNT + TGFb vs. siNT	3	< 0.0001	****
		siNT + TGFb vs. siSolo#1	3	< 0.0001	****
		siNT + TGFb vs. siSolo#2	3	< 0.0001	****
	F	GFP vs. GFP-Solo	3	0.0065	**
		GFP vs. GFP-Solo L1217E	3	< 0.0001	****
		GFP-Solo vs. GFP-Solo L1217E	3	< 0.0001	****
	F	GFP vs. GFP-Solo	3	0.0026	**
		GFP vs. GFP-Solo L1217E	3	0.6212	n.s.
		GFP-Solo vs. GFP-Solo L1217E	3	0.0358	*
**Figure S5**	-	RhoA: GFP-Solo vs. GFP-Solo Y242F	3	0.4116	n.s.
		RhoB: GFP-Solo vs. GFP-Solo Y242F	3	0.1226	n.s.
**Figure 3**	B	GFP vs. GFP-Solo	4	< 0.0001	****
		GFP vs. GFP-Solo Y242F	4	< 0.0001	****
		GFP vs. GFP-Solo L1217E	4	< 0.0001	****
		GFP-Solo vs. GFP-Solo Y242F	4	0.0002	***
		GFP-Solo vs. GFP-Solo L1217E	4	< 0.0001	****
		GFP-Solo Y242F vs. GFP-Solo L1217E	4	0.0985	n.s.
	D	GFP vs. GFP-Solo	3	< 0.0001	****
		GFP vs. GFP-Solo Y242F	3	< 0.0001	****
		GFP vs. GFP-Solo L1217E	3	0.9105	n.s.
		GFP-Solo vs. GFP-Solo Y242F	3	0.5109	n.s.
		GFP-Solo vs. GFP-Solo L1217E	3	< 0.0001	****
		GFP-Solo Y242F vs. GFP-Solo L1217E	3	< 0.0001	****
	F	GFP vs. GFP-Solo	3	0.0228	*
		GFP vs. GFP-Solo Y242F	3	0.0143	*
		GFP vs. GFP-Solo L1217E	3	0.9482	n.s.
		GFP-Solo vs. GFP-Solo Y242F	3	0.9886	n.s.
		GFP-Solo vs. GFP-Solo L1217E	3	0.0382	*
		GFP-Solo Y242F vs. GFP-Solo L1217E	3	0.0241	*
**Figure S7**	-	GFP vs. GFP-Solo	3	0.0047	**
		GFP vs. GFP-Solo Y242F	3	< 0.0001	****
		GFP vs. GFP-Solo L1217E	3	0.0736	n.s.
		GFP-Solo vs. GFP-Solo Y242F	3	0.182	n.s.
		GFP-Solo vs. GFP-Solo L1217E	3	< 0.0001	****
		GFP-Solo Y242F vs. GFP-Solo L1217E	3	<0.0001	****
**Figure 4**	C	siNT vs. siSolo#1	3	0.0041	**
		siNT vs. siSolo#2	3	0.0015	**
	F	siNT vs. siSolo#1	3	0.0026	**
		siNT vs. siSolo#2	3	0.0461	*

	H	siNT vs. siSolo#1	3	0.0384	*
		siNT vs. siSolo#2	3	0.0216	*
	I	MDA-MB-231: siNT vs. siSolo#1	3	0.036	**
		MDA-MB-231: siNT vs. siSolo#2	3	< 0.0001	****
		BT549: siNT vs. siSolo#1	3	< 0.0001	****
		BT549: siNT vs. siSolo#2	3	< 0.0001	****
		HS578T: siNT vs. siSolo#1	3	0.0007	***
		HS578T: siNT vs. siSolo#2	3	< 0.0001	****
**Figure S9**	B	siNT vs. siSolo#1	3	0.0233	*
		siNT vs. siSolo#2	3	0.0121	*
**Figure S11**	B	siNT vs. siSolo#1	3	0.1839	n.s.
		siNT vs. siSolo#2	3	0.7414	n.s.

Table S2: List of oligonucleotides used for cloning and site directed mutagenesis, related to STAR methods

Plasmid		Sequence
GFP-Solo	fw	5’-CCGGAATTCTGAGCCTGAGCCAGTGGAGGAC-3'
	rev	5’-CCGGAATTCACAGAGGCGTGGTGGGGTC-3’
GFP-Solo L1217E QC	fw	5’-CTCGGTATGGGCGGGAGCTGGAGGAGCTCCTG-3’
	rev	5’-CAGGAGCTCCTCCAGCTCCCGCCCATACCGAG-3’
GFP-Solo Y242F QC	fw	5’- GAGGGCGAGTTTGTGGAGCTGTTAGAGG -3’
	rev	5’- CTCCACAAACTCGCCCTCAGGTCCTTC -3'
Solo TRE3G	fw	5’-GCTTGCGTTGGATCCCTCGAATGGTGAGCAAGGGCGAG-3’
	rev	5’-GGTATCGATAAGCTTGATAATTCTTAATTCACAGAGGCGTGGTGGG-3’
mEos3.2 amp	fw	5’- GGTTTTGGCAGTACATCAATGGGC-3’
	rev	5’-GATATGAATTCGAGTCGCGGCCGCTTATCG-3’
