# Supplementary material for: Reciprocal regulation of Solo and Src orchestrates Src trafficking to promote mesenchymal cell migration
Source: iScience. 2025 May 9;28(6):112618. doi: 10.1016/j.isci.2025.112618 (PMC12152665; doi:10.1016/j.isci.2025.112618)

Fig 1B

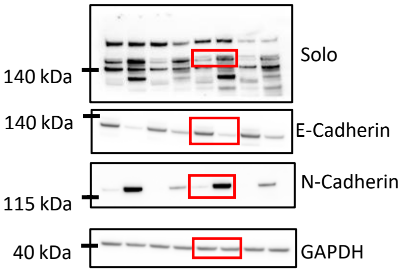

Fig 1C

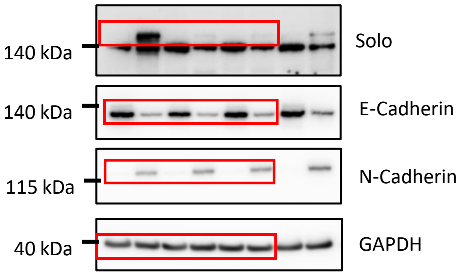

Fig 2A

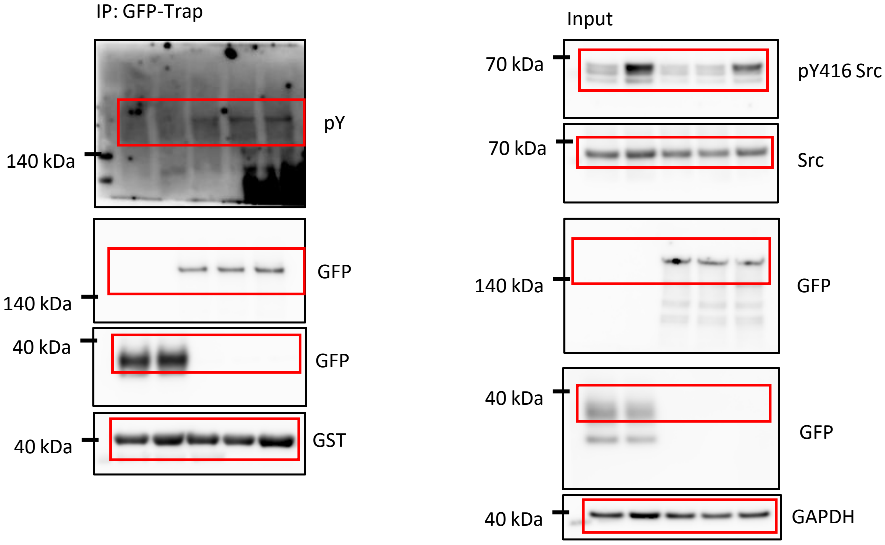

Fig 2B

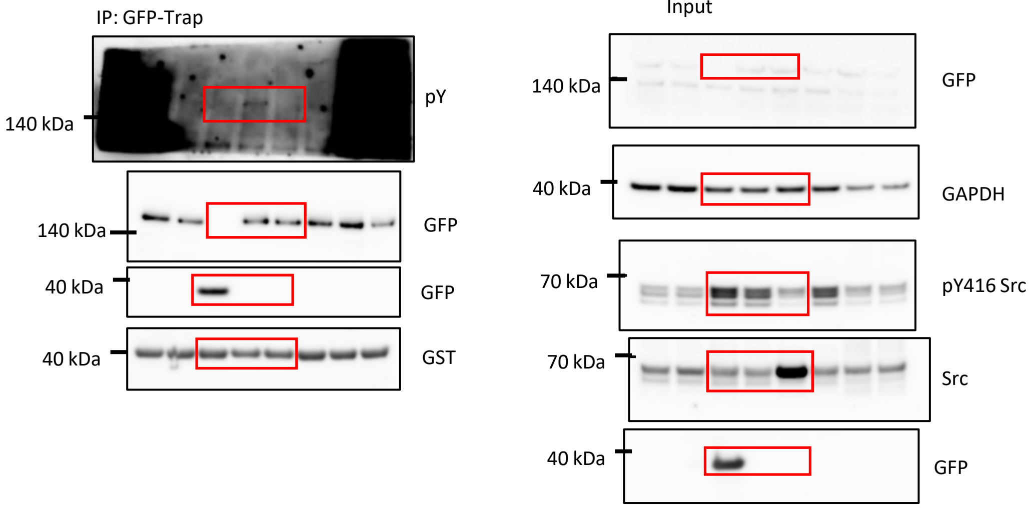

Fig 2C

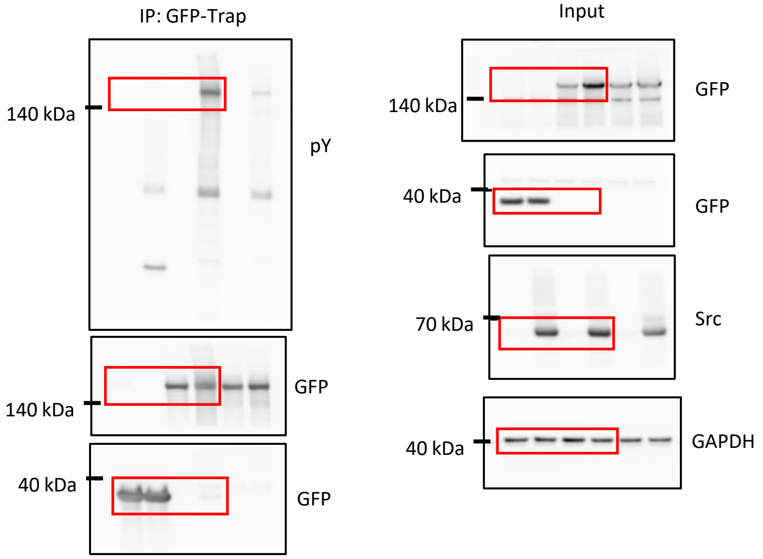

Fig 2D

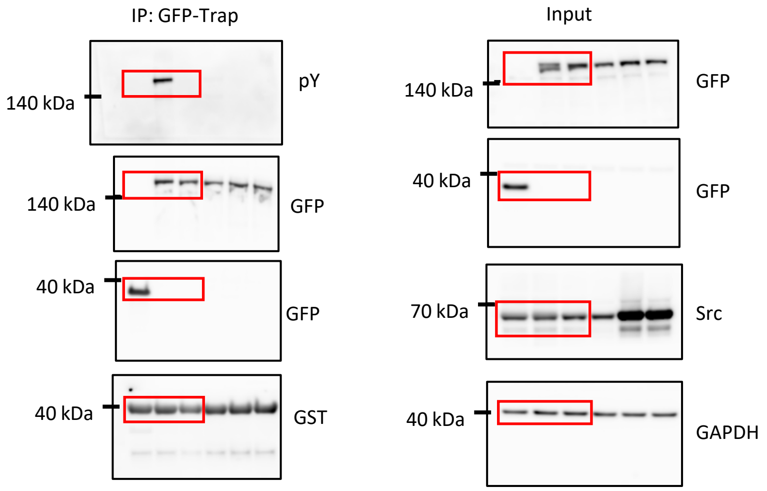

Fig 2E

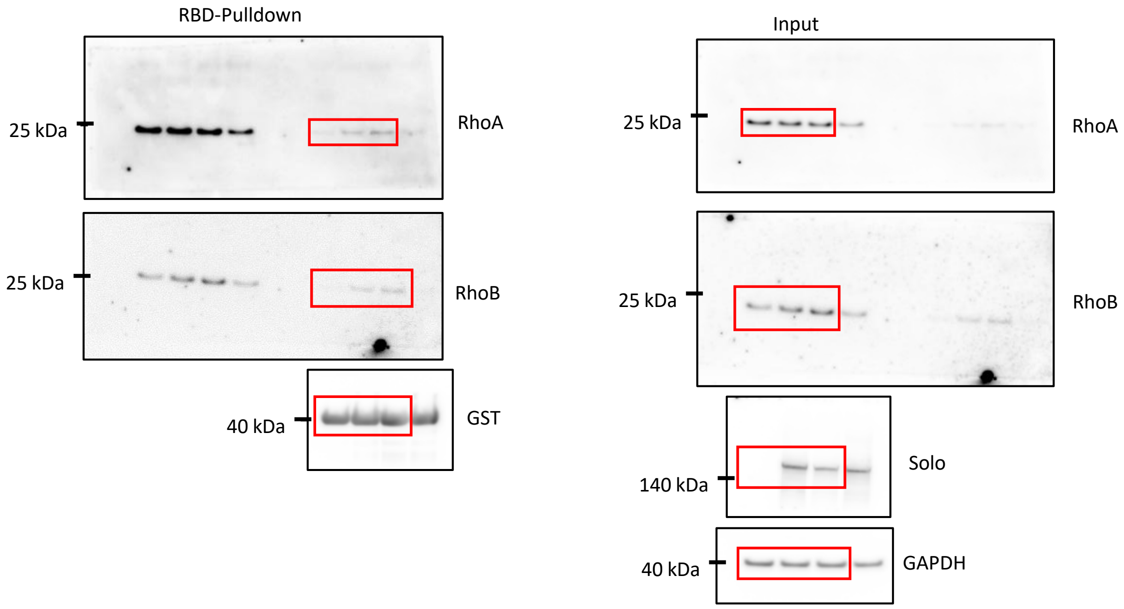

Fig 3A

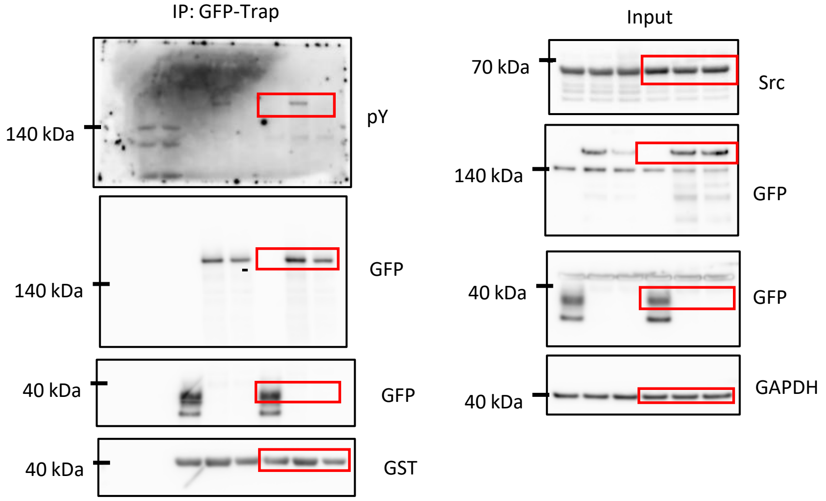

Fig S2

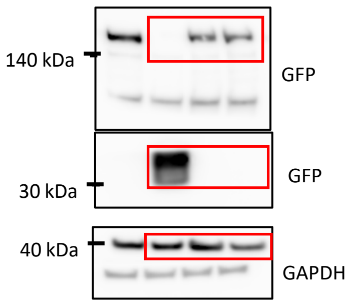

Fig S6

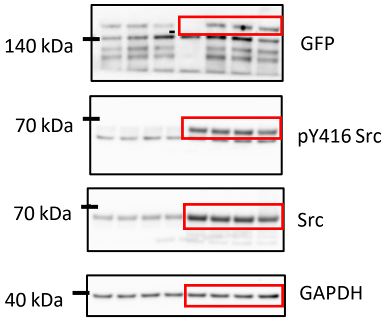

Fig S8

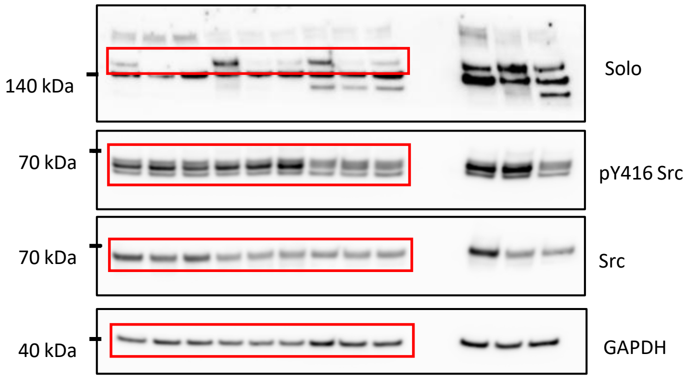

Fig S10A

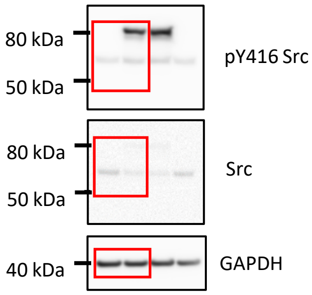

Fig S10B

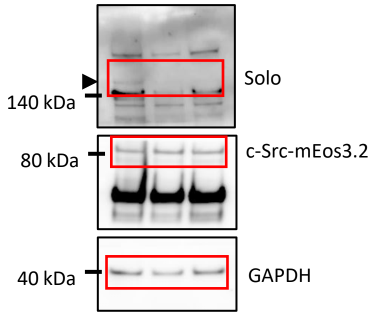

Supplement: Document S2. Uncropped western blots [file mmc2.pdf]
